# Recruitment of multi-segment genomic RNAs by Bluetongue virus requires a preformed RNA network

**DOI:** 10.1093/nar/gkae404

**Published:** 2024-05-20

**Authors:** Po-yu Sung, Jody E Phelan, Dongsheng Luo, Raghavendran Kulasegaran-Shylini, Patrick Bohn, Redmond P Smyth, Polly Roy

**Affiliations:** Department of Infection Biology, London School of Hygiene and Tropical Medicine, London, UK; Department of Infection Biology, London School of Hygiene and Tropical Medicine, London, UK; Department of Infection Biology, London School of Hygiene and Tropical Medicine, London, UK; Department of Infection Biology, London School of Hygiene and Tropical Medicine, London, UK; Helmholtz Institute for RNA-based Infection Research (HIRI), Würzburg, Germany; Helmholtz Institute for RNA-based Infection Research (HIRI), Würzburg, Germany; Faculty of Medicine, University of Würzburg, Würzburg, Germany; Department of Infection Biology, London School of Hygiene and Tropical Medicine, London, UK

## Abstract

How do segmented RNA viruses correctly recruit their genome has yet to be clarified. Bluetongue virus is a double-stranded RNA virus with 10 segments of different sizes, but it assembles its genome in single-stranded form through a series of specific RNA–RNA interactions prior to packaging. In this study, we determined the structure of each BTV transcript, individually and in different combinations, using 2′-hydroxyl acylation analysed by primer extension and mutational profiling (SHAPE-MaP). SHAPE-MaP identified RNA structural changes during complex formation and putative RNA–RNA interaction sites. Our data also revealed a core RNA-complex of smaller segments which serves as the foundation (‘anchor’) for the assembly of a complete network composed of ten ssRNA segments. The same order of core RNA complex formation was identified in cells transfected with viral RNAs. No viral protein was required for these assembly reactions. Further, substitution mutations in the interacting bases within the core assemblies, altered subsequent segment addition and affected virus replication. These data identify a wholly RNA driven reaction that may offer novel opportunities for designed attenuation or antiviral therapeutics.

## Graphical Abstract

**Graphical Abstract:**
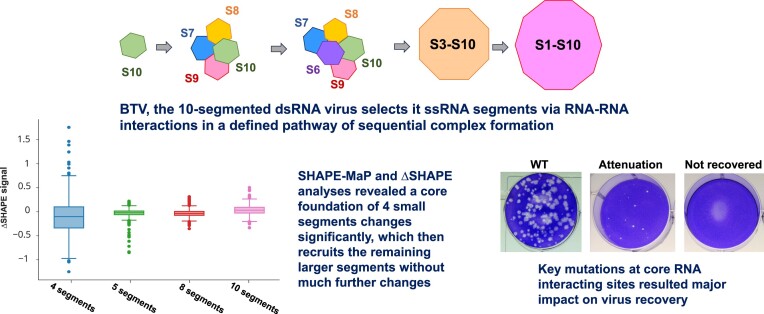

## Introduction

Genome selection and packaging are fundamental processes in virus life cycles that are crucial to produce new infectious particles. For single-stranded RNA (ssRNA) viruses, it is commonly accepted that genome selection is initiated through a specific interaction between viral proteins and the genomic RNA. The discrimination between cellular and viral RNA is achieved by recognition of well-defined *cis*-acting RNA sequences or structures within the viral genome (1–8). An additional challenge arises for RNA viruses with multi-segmented genomes, where genome packaging requires the incorporation of at least one copy of each segment to ensure of the progeny particles are infectious. For viruses with segmented ssRNA genomes, such as the eight-segmented Influenza virus, each RNA segment interacts individually with nucleocapsid proteins before or during genome packaging (9–12). However, the precise packaging mechanism remains unclear, although it likely involves RNA-protein, protein-protein and RNA–RNA interactions. For viruses with segmented double-stranded RNA (dsRNA) genomes, the mechanism must differ since dsRNA segments are not packaged but are synthesised within a procapsid by the viral polymerase from ssRNA templates that are already packaged (13,14). Current data suggests specific ssRNA structures and intermolecular interactions are likely to guide the assembly of a packaging competent ssRNA complex that is subsequently selected into capsid structures (15–17).

To elucidate the RNA–RNA interactions and packaging mechanisms for dsRNA viruses, we used Bluetongue virus (BTV), a segmented dsRNA virus, as a model virus. BTV is an animal pathogen and is the prototype of the Orbivirus genus (29 related serogroups; 140 members) in the family *Reoviridae* (15 distinct genera), which consists of viruses that infect a wide range of hosts including humans (e.g. rotavirus), animals and plants (18). All members are nonenveloped viruses with icosahedral capsids, made up of concentric protein layers. A unique feature of this family members is that their genomes are multipartite dsRNAs (up to 12 segments), the replication and packaging of which remains subject to investigation.

For BTV, the outer capsid is made up of VP2, the cell surface binding protein, and VP5, the cell membrane penetration protein. Both proteins are attached directly to the next layer, made up of VP7, which forms the outer layer of the inner capsid. Underneath the VP7 layer is another layer formed by VP3, which surrounds the viral polymerase complex (PC) of three enzymatic proteins, VP1, VP4 and VP6 and the genome of ten RNA segments (S1–S10). BTV RNA segments vary in sizes (3.95–0.8 kb), but each shares common short 5′ and 3′ untranslated regions (UTRs) of variable length, including highly conserved hexanucleotides at either end (19).

Upon entry into the cytoplasm, the outer capsid layer is detached to release the double-layered inner capsid. The inner capsid does not disassemble further, but triggers the transcription of each dsRNA segment, synthesizing 10 positive single-stranded RNAs (+ssRNAs) repeatedly that are extruded through specific capsid channels into the cytoplasm (20–23). These transcripts serve as templates for viral protein synthesis, and also later act as templates for progeny genomic dsRNA formation. The molecular mechanisms of ssRNA packaging and replication are not precisely known. Using sequential *in vitro* transcription and translation of BTV +ssRNAs, we showed that the 10 +ssRNA segments of BTV associate with the PC prior to encapsidation by VP3, followed by the addition of the VP7 layer leading to the formation of a stable double-layered inner capsid (24). Further, we showed that the packaged +ssRNA segments within these cell-free assembled particles, serve as templates for dsRNA synthesis, producing a complete equimolar set of genome segments (14). Notably, we discovered that the +ssRNA transcripts assemble spontaneously into an RNA interaction network in the absence of any viral protein prior to being packaged into the assembling particles (14,17). Correspondingly, the disruption of certain RNA–RNA interactions could inhibit packaging and virus infectivity (15,25,26).

The RNA–RNA interaction observed for co-synthesised ssRNA mixtures could be predicted by a bespoke bioinformatics model, and the specific interactions among the smaller RNA segments were validated experimentally (15). The concept of multipartite genomic RNA segments selection via an RNA network prior to or during packaging is appealing as it obviates the need for the assembling virus to recognize multiple RNA molecules. However, direct experimental evidence for the points of contact among the interacting segments is lacking. Neither the sequences nor any structural changes that accompany them during the formation of the complex is known, and whether the complex forms by sequential steps or by all segments coalescing together at one time is also unclear.

In this study, we investigated RNA structural changes during the assembly of a complete set of ten BTV ssRNA transcripts and addressed whether a defined sequential order was required. For this, we used selective 2'-hydroxyl acylation analysed by primer extension and mutational profiling (SHAPE-MaP) to define the RNA structure of each BTV segment at nucleotide resolution (27–30). Each BTV transcript, individually and in different combinations, was subjected to SHAPE-MaP. Comparisons of the SHAPE-MaP profiles of each segment individually and in the presence of different combinations of RNA segments, revealed structures within each RNA segment and unique stable ‘core’ RNA network consisting of only a few RNA segments, that acts as the anchor for the selection of the remaining segments. Similar profiles of RNA–RNA interactions and complex formation were found in the cell's cytoplasm following transfection of synthetic fluorophore labelled BTV transcripts confirming that their formation is independent of viral protein interaction. Further, using a combination of nucleotide substitutions, a cell-free *in vitro* RNA packaging assay (CFA) (14) and the *in vivo* BTV reverse genetics system (RG) (31) we validated the critical structures of different core RNA segments as being required for the generation of infectious BTV.

## Materials and methods

### Virus, plasmids, RNA transcript synthesis and cell culture

BTV-1 (GenBank accession numbers FJ969719 to FJ969728) was used for this study. The 10 genome segments were firstly cloned into pUC19 plasmid with T7 promoter and RNA transcripts sequentially prepared using the mMACHINE T7 transcription kit (Thermo). BSR cells (BHK-21 subclone, ATCC^®^ CCL10™) were grown in Dulbecco's modified Eagle's medium (DMEM) (Sigma) supplemented with 5% fetal bovine serum (Sigma) at 37°C with 5% CO_2_.

### Mutagenesis

Mutations introduced into BTV plasmids by site-directed mutagenesis using the primers listed in Supplementary Table S1. Mutations were confirmed with Sanger sequencing.

### SHAPE-MaP

BTV genomic transcripts were modified either individually or in different combinations of complexes. The complex formation procedure was described previously (17). In brief, an equimolar ratio of BTV RNA transcripts was denatured at 80°C for 1 min, immediately chilled and then mixed with folding buffer (50 mM sodium cacodylate pH 7.5, 100 mM KCl and 10 mM MgCl_2_) for 30 min at 30°C. SHAPE modification was performed as described (30,32). In brief, 2 μg of RNA was treated with 100 nM 1-methyl-7-nitroisatoicanhydride (1M7) for 5 mins at 30°C after the folding reaction. Two additional control reactions were performed: an untreated control, where RNA was incubated with 5 μl of dimethyl sulfoxide (DMSO), and a denatured control, where RNA was incubated at 95°C for 2 min and then treated with 100 nM 1M7 at 95°C. RNAs were then purified, and cDNAs were generated with SuperScript II (Thermo Fisher Scientific) using MnCl_2_ containing reverse transcriptase buffer (1 mM deoxynucleotide triphosphates, 10mM dithiothreitol, 50 mM Tris–HCl [pH 8.0], 75 KCl, and 50 mM MnCl_2_). Second strand synthesis was performed using the NEBNext mRNA second strand synthesis module (NEB) according to the manufacturer's protocol. The DNA was then fragmented, tagged, amplified, and barcoded using a Nextera XT DNA library preparation kit (Illumina) according to the manufacturer's directions. Sequencing was then performed on a SP flow-cell on a NovaSeq sequencer (Illumina).

### SHAPE data processing and analysis

SHAPE probing was performed in duplicate for all samples, analysed with ShapeMapper2 (v2.1.5) using the default parameters with raw Fastq data for the denatured, untreated and treated datasets together with appropriate reference sequences. The normalized mean reactivity (Norm profile) of those replicates was used for further data analysis. ΔSHAPE reactivity values were calculated by subtracting the values of smaller complex from the larger complex producing a delta (Δ) value. This was performed with the deltaSHAPE software (v1.0) using the no-show and all parameters to produce map files. Outputs were plotted using the Plotly (Python) library. RNA structure predictions were calculated using the rf-fold module of RNA Framework (33). The match between SHAPE reactivities and the predicted RNA secondary structure was calculated using the receiver operator characteristic area under the curve (ROC-AUC) score function of the Sklearn python library. The similarity of shape reactivity profiles, and hence structure, between segments within different complexes was calculated by principal component analyses (PCA) using the PCA function from the Sklearn package (v1.3.0). To quantify the overall structural change within a segment during complex formation we calculated the mean absolute difference per position between two samples. Specifically, we calculated the average absolute difference between the single segment compared to the S7–S10 complex, the S7–S10 compared to S6–S10 complex and the S6–S10 compared to S1–S10 complex. To quantify the similarity of reactivity profiles between all complexes we calculated the pairwise Pearson correlation. Calculations were performed with NumPy (Python), and figures were generated with Plotly (Python).

### Fluorescence *in-situ* hybridization chain reaction

RNA fluorescence *in-situ* hybridization chain reaction (HCR) was performed according to published protocols with some modifications (34,35). The HCR is a two-step *in-situ* hybridization technique. In the first step, a mixture of probes with initiator nucleotide tails (30 oligonucleotides per RNA segment) which encompass the sequences of negative sense viral RNA segments thereby targeting the positive viral RNAs, referred to as ‘Target’ probe. In the second step, an isothermal enzyme-free polymerization method was employed that uses two special detection hairpin nucleotide probes with fluorescent tags (36): H1 and H2 (probes were synthesized by IDT Inc.; Supplementary Table S2). These hairpin molecules are present in solution until the introduction of the initiator strands of the target probes, which trigger a cascade of hybridization events, yielding nicked double helices analogous to alternating copolymers.

Each viral ssRNA segment was transfected at 100 ng per well in a 12-well plate using X-TremeGENE HP DNA Transfection Reagent (Roche), in accordance with the manufacturer's instructions. The cells were incubated at 37°C with 5% CO_2_ for 16 h before being washed once with ice-cold PBSM (1 × PBS, 5 mM MgCl_2_), followed by fixation with 4% paraformaldehyde in PBSM for 10 min at room temperature (RT). After a brief wash with ice-cold PBSM, the cells were permeabilized with 0.1% Triton X-100 in PBSM for 10 mins at room temperature. The cells were then washed with PBSM and incubated in Pre-hybridization buffer which includes 2 × SSC (300 mM NaCl, 30 mM sodium citrate) with 10% formamide for 20 min before hybridization. The hybridization buffer was preprepared with 10% dextran sulphate (Cayman Chemical Company), 2 mM vanadyl ribonucleoside complexes (VRC, New England BioLabs), 0.02% RNase free BSA, 50 mg *E. coli* tRNA, 10% formamide and 2 × SSC, then mixed with the oligonucleotide probes, to a final concentration of 5ng/μl for the first hybridization step, and 60 nM of hairpin DNA pairs for the second hybridization step.

The first hybridization was carried out in a humidified chamber at 37°C for a minimum of 5 h or overnight. The samples were then washed twice with 10% formamide in 2 × SSC for 30 min at 37°C. Prior to the second hybridization, the annealing step for the hairpins was performed in a thermocycler from 95°C to 25°C, the two HCR hairpins were then annealed separately. The second hybridization was then performed at 25°C or at room temperature for 5 h to overnight in a humidified chamber. Nuclear staining using 0.5 mg/ml of Hoechst 33342 (Thermo Fisher Scientific) was then performed and the coverslips were mounted in ProLong Gold antifade mounting media (Invitrogen). The cured samples were subjected to microscopy examination.

### Image acquisition and analysis

Cells were placed on a Zeiss LSM 880 microscope equipped with a 63×, 1.4 numerical aperture oil-immersion objective (Zeiss) and a digital camera. Quantification of the fluorescent photographs was performed at the same threshold and adjustment of contrast. Images were exported as tiff format and analysed with Fiji.

### Reverse genetics and plaque assay

Mutant RNA segments were produced from the mutant T7 cDNA, together with the other 9 BTV genome segments to transfect BSR cells, as described by Boyce *et al.* (31). Cytopathic effect (CPE) was monitored after 3 days, and the mutations in the recovered viruses were confirmed by RT-PCR and sequencing. WT and mutant viruses were diluted, applied to BSR cell monolayers at a multiplicity of infection (MOI) of 0.01–0.1, and overlayed with Avicel as described by Matrosovich *et al.* (37). After 3 days, the cells were fixed with formaldehyde and stained with crystal violet, the plaque size monitored.

### *In vitro* cell-free RNA packaging assay

The BTV *in vitro* cell-free RNA packaging assay has been described previously (14). Briefly, the 10 segments of ssRNA, either with mutant segment or all wild type, were incubated with *in vitro* expressed VP1, VP4 and VP6 for 30 min, followed by the addition of VP3 and VP7 sequentially with a 1.5 h incubation after each addition. The samples were then subjected to a 15–65% sucrose gradient, the 40% sucrose band was collected, treated with RNase One to remove the unpackaged RNAs, and the packaged RNA was quantified by qRT-PCR, detecting S6 as representative, as previously described (17).

## Results

### Identification of BTV RNA secondary structures changes during initial complex formation

**Figure 1.**
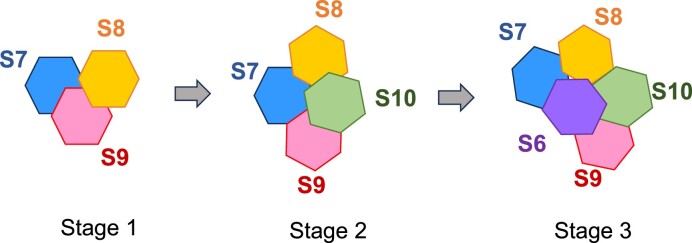
A schematic flow chart of computer predicted BTV RNA complex formation. The three stages of BTV S6-S10 RNA segment assembly predicted by computer analysis (15) is presented as a cartoon.

RNA–RNA interactions and complementarity between the segments are considered the key mechanism for BTV to recruit and assemble the correct set of genomic segments (17,26). Previously, based on a series of biochemical data of BTV RNA–RNA interactions assays, we modelled the dynamic association of five smaller BTV RNA segments (S6–S10) (15). Using this bespoke computer model, we predicted that the interactions among these segments occur in three stages, an initial S7 + S8 + S9 complex of three segments (Stage 1), then its association with the smallest S10 segment (Stage 2), and subsequently its association with S6, which is slightly larger than the S7 segment (Stage 3) (Figure 1). At each stage, specific interacting sites were predicted based on the energy threshold and further, several putative interacting sites were confirmed by biological data. However, it was not computationally tractable to reveal the exact changes occurring in each segment during the formation of each complex or to model the association of the larger segments.

**Figure 2.**
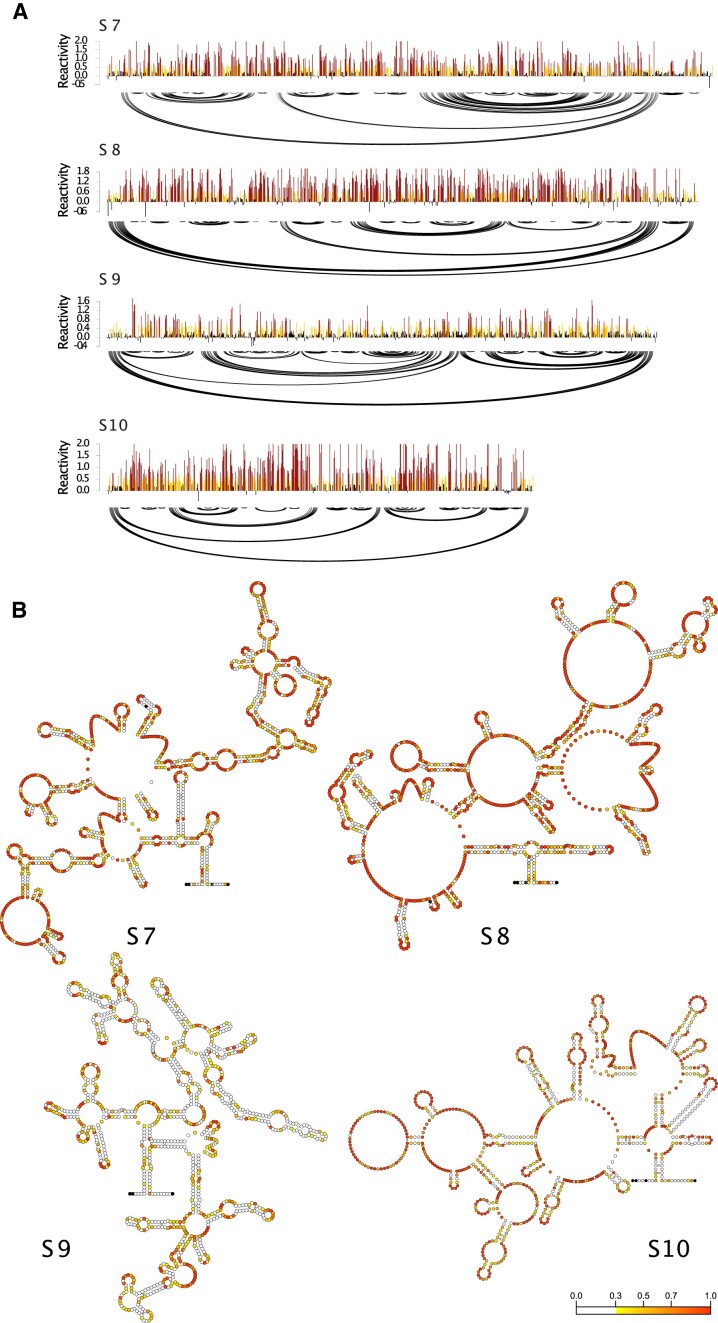
The SHAPE profiles of BTV S7, S8, S9 and S10. (**A**) SHAPE-MaP data and secondary structure predictions for BTV segments S7–S10. Models were obtained using 1M7 reactivities as soft constraints for in silico folding using the rf-fold module of RNAFramework. 1M7 reactivities for each nucleotide position are show in the bar chart using a continuous colour scale (0 white, 0.3 yellow, 0.7 orange, 1 red). Arc plots show consensus structure. (**B**) S7–S10 consensus (ensemble) secondary structure, generated by RNAfold integrating SHAPE data.

To address these, we used SHAPE-MaP to interrogate RNA structure at single nucleotide resolution and with high accuracy. For this, *in vitro* transcribed RNA of each segment (S7–S10) was individually probed with 1M7, reverse-transcribed to cDNA and sequenced to identify the changes corresponding to regions of ssRNA. SHAPE-MaP data of two independent experiments for S7–S10 showed almost identical chemical reactivity profiles indicating that BTV segments assemble into unique reproducible conformations. Based on the SHAPE-MaP data, we then generated RNA secondary structure models using RNA framework, revealing the formation of extensive secondary structure within each RNA segment (Figure 2 and Supplementary Figure S1). Notably, we calculated the correlation co-efficient between the SHAPE reactivities and the RNA structure predicted in the absence or presence of SHAPE-MaP data. For this, we used the ROC-AUC score, which is commonly used to statistically evaluate the correlation of SHAPE reactivities with single stranded regions. A score of 0.5 signifies a random association of the two variables, whereas 1 indicates a perfect match. Without the inclusion of SHAPE data, the ROC-AUC scores were 0.71. 0.66, 0.67 and 0.68 for S10, S9, S8 and S7, respectively. With SHAPE data, the ROC-AUC scores increased to 0.83, 0.72, 0.81 and 0.79 for S10, S9, S8 and S7, respectively. Therefore, the inclusion of SHAPE data substantially enhances the correlation between the structure predictions and experimental data, suggesting our structural models are an accurate representation of the folded molecule.

**Figure 3.**
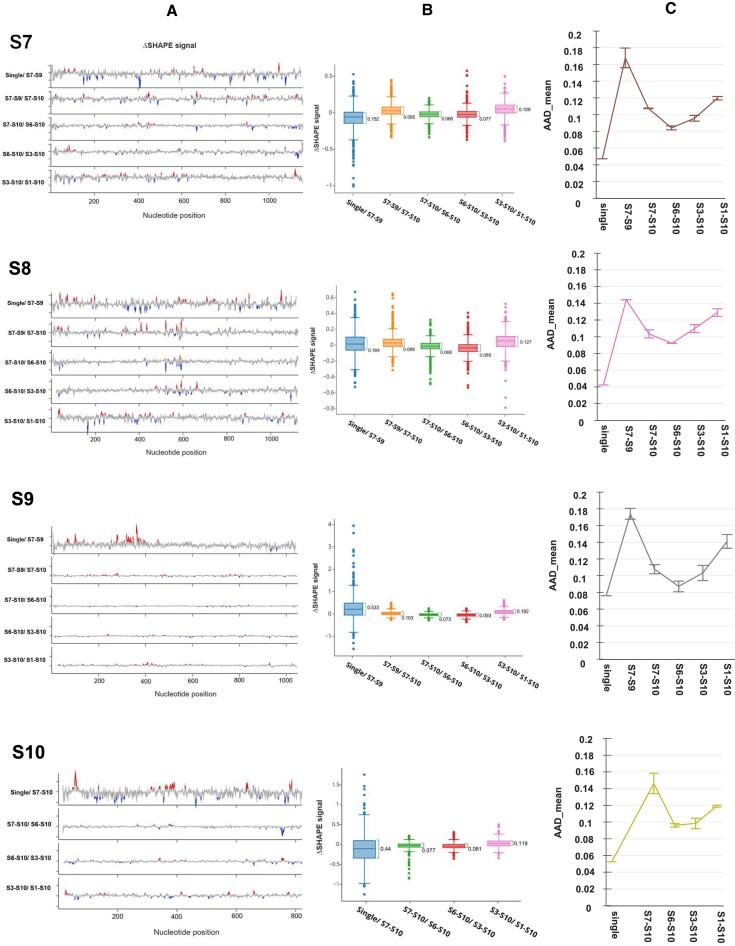
The ΔSHAPE profiles of S7–S10 in different stages of RNA complex. (**A**) Line plots showing the ΔSHAPE values of S7–S10 in single form and in different complexes (S7–S9, S7–S10, S6–S10, S3–S10 and S1–S10). These values indicate the difference in SHAPE values between single and primary complex or two different complexes, where the reactivity values from one complex are subtracted from the other. The red peaks indicate increases of SHAPE signals in the larger complexes and blue valleys indicate decreases. (**B**) Boxplots showing the distribution of values displayed in (A). A wider distribution indicates more extreme values between the two complexes compared. The inter-quartile ranges (Q3 minus Q1) are indicated. (**C**) Average absolute deviation of SHAPE reactivity profiles during complex formation. Shown is the average per position reactivity change as the size of the complexes increase. Error bars indicate the standard deviation of the average per position reactivity change.

To identify structural changes in each segment during the Stage 1 complex formation, S7, S8 and S9 were incubated together and then subjected to SHAPE-Map analysis (38). The SHAPE reactivity measurements of each segment in the complex were then compared with the individual RNA segments. Using ΔSHAPE, which compares chemical probe reactivity measurements between two conditions after considering measurement errors at each nucleotide, statistically significant changes in SHAPE reactivity were identified, which both increased and decreased SHAPE reactivity, indicating extensive secondary structure rearrangements occurred upon complex formation. Globally, these ΔSHAPE changes were enriched in single stranded regions (Figure 3A, Supplementary Figure S2). The data confirmed that the secondary structures among the segments were altered upon complex formation and possibly, new RNA–RNA interactions were formed. The structural changes upon Stage 1 complex formation were extensive, as reflected in the large inter-quartile ranges (IQRs) of 0.152, 0.164 and 0.533 for segments S7, S8 and S9, respectively (Figure 3B). To further confirm this, we performed average absolute deviation analysis, which showed consistency with the IQR data, demonstrating high deviation from the single segment data to that obtained with the Stage 1 complex (Figure 3C). Further, two independent experiments showed highly similar SHAPE patterns in the complex. Specifically, the ΔSHAPE profile of S7 suggests distinct interactions with S8 and S9 in the complex. At least 199 of the 1156 nucleotides of S7, dispersed over the whole segment, were considerably different in the Stage 1 complex compared to the individual segment (Figure 3). Indeed, the ΔSHAPE profile for S7 revealed numerous protections in a previously mapped single stranded loop, suggestive of new intermolecular interactions with S8 and S9 in the complex. Similarly, S8 and S9 exhibited significant ΔSHAPE changes upon complex formation, which were distributed evenly across each segment. For S8, 229 of the 1125 nucleotides changed, higher than the other segments (Figure 3). Interestingly, these changes were enriched at the 5’ end of the segment, between nts20–100, and the region spanning nts340–450. In contrast, S9 exhibited fewer changes, with only 111 of the 1049 nucleotides significantly changing in the complex, with the majority of those changes located between nts275–397(Figure 3A, Supplementary Figure S2).

To examine if the previously predicted interacting sites coincide with the newly identified changes by SHAPE-MaP analysis, we compared each segment of the Stage 1 complex of S7–S9. Of the computer predicted 6 interacting sites in S7 (nts172–187, nts233–242, nts377–388, nts478–489, nts701–710 and nts119–1133 respectively), four overlapped the regions of significant ΔSHAPE change. Of the other two, one (nts377–388) is in the vicinity of a significant ΔSHAPE site (nts393–411), while the other (nts478–489) is located between two ΔSHAPE sites (nts473–475 and nts493–497). These data suggest that previous predictions of RNA interaction sites were broadly consistent with the chemical data obtained for S7 but lacked the same level of definition. The similarity between the two methods was less obvious for S8 and S9. There were three predicted sites in S8, but none overlapped the actual data, although two sites (nts85–98 and nts456–469) were located close to the sites identified by ΔSHAPE (nts100–107 and nts448–451). Together, the modelling predictions of interacting sites in mixtures of RNA segments indicated sites which, in some cases, matched those determined experimentally and in other cases gave locations close to those obtained by experimentation.

We next investigated if the addition of S10 would modify the SHAPE profiles of the smaller segments when they form the S7–S10 complex (Stage 2). We found that indeed S10 induced substantial structural changes in each of the other three segments, with 199, 229 and 111 nucleotides demonstrating significant ΔSHAPE changes in S7, S8 and S9 respectively due to complex formation (Figure 3, Supplementary Table S3). As expected, extensive structural rearrangements within S10 also occurred during Stage 2 complex formation, as indicated by a high IQR of 0.44 in the ΔSHAPE analysis (Figure 3). Out of 822 nucleotides of S10, 171 were altered, reflecting structural rearrangements as well as interactions between S10 and the three other (S7-S9) segments. Multiple clusters of longer sequences were identified along the S10 transcript, such as nts47–61, nts21–131, nts374–392, nts534–545 and nts773–787. Despite significant SHAPE reactivity changes in all segments, the magnitudes of these changes were lower than at Stage 1, with IQRs of 0.095 (S7), 0.089 (S8) and 0.104 (S9) and higher in S9 than in S7 or S8 (Figure 3B). When S6 was added to form the Stage 3 complex (S6–S10), the IQRs were not significant (0.066–0.077), which was also confirmed by the average absolute deviation values, suggesting less impact by S6 on the smaller segments (Figure 3B, C). These data are consistent with the S7–S10 complex serving as a robust foundation for the sequential recruitment of the larger segments with little further structural rearrangement.

### The *trans-* and *cis-*interactions in the complete BTV genome complex

We next analysed the SHAPE-MaP data of the two larger complexes, namely, the S3–S10 complex (addition of the three larger segments) and S1–S10 (the complete set of ten segments) (Figure 3A). Addition of the larger segments to the S6–S10 complex showed a relatively minor impact on the SHAPE-MaP reactivities of S7–S10, when compared to the Stage 1 complex (Figure 3B, C). This was confirmed by a correlation analysis of the SHAPE data among different complexes, which showed extensive similarity between the three different complexes, but lower similarity between any complex and any individual segment (Supplementary Figure S3). These results demonstrate that the initial interaction between the smaller segments is concomitant with large structural rearrangements and that addition of the large segments caused little further structural change, consistent with the Stage 1 complex serving as the foundation for the assembly of larger complexes.

**Figure 4.**
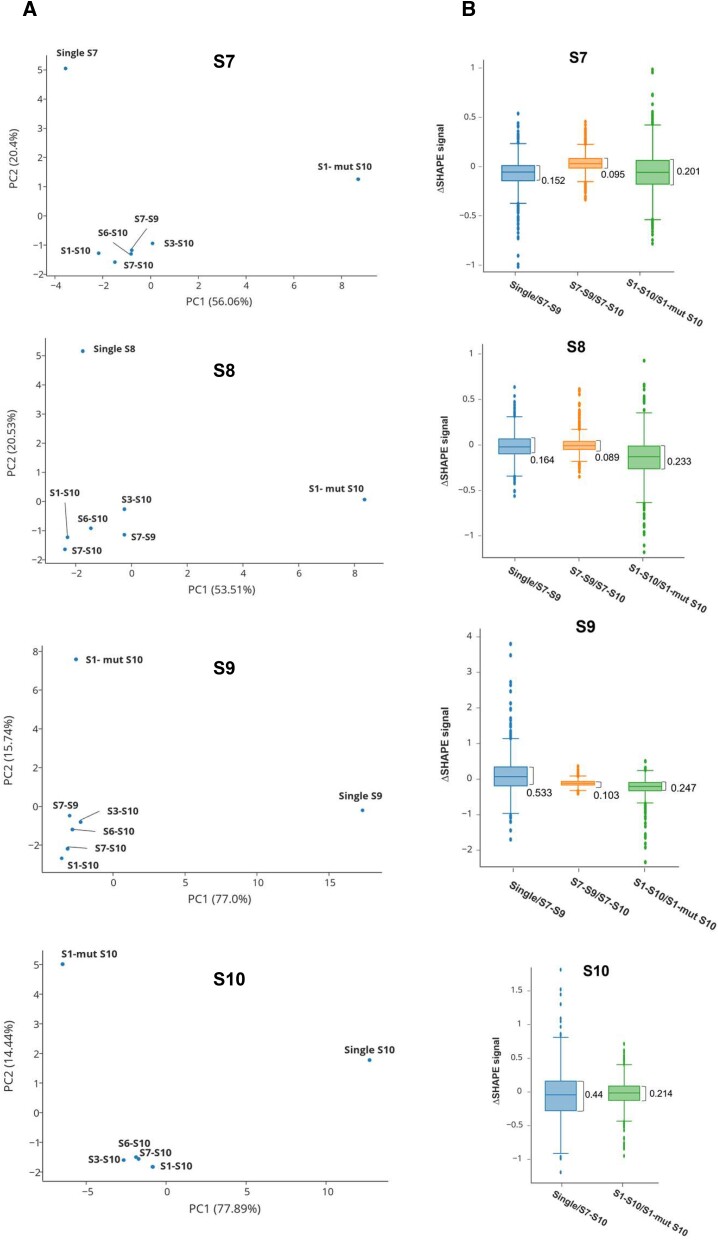
The ΔSHAPE profiles change when mutant S10 is included. (**A**) Plots of the principal component analysis using the SHAPE-MaP reactivity values as features. Reactivity profiles from complexes that have a greater degree of similarity cluster near each other. Complexes that cluster separately have differential profiles. (**B**) Boxplots showing the distribution of values as described in Figure 3. The inter-quartile ranges (Q3 minus Q1) are indicated.

It has been reported that S10 plays a particular role in genome packaging (14,16,17). As S10 also affected the S7–S9 complex extensively, we examined if a key mutation in S10 would affect the SHAPE-MaP structural profile of S1–S10 complex. We used the S10 mutant previously shown not to engage *in vitro* RNA complex formation (15). The mutant has changes in nts21-41 (5’-GCUAUCCGGGCUGAUCCAAA-3’ → 5’-G**U**U**GAGU**GG**CU**U**A**AU**A**CA**G**A-3’) which disrupt S6–S10 complex formation when assayed by gel electrophoresis mobility shift assay (EMSA), and also to be lethal to infectious virus recovery by reverse genetics (15). We incubated mutant S10 ssRNA together with the other nine wild-type ssRNA segments (S1–S9) to form the complete genome complex followed by SHAPE-MaP analysis. When the data was compared with the wild-type S1–S10 complex, the SHAPE signals were significantly altered not only in S10, but also in the other segments (Supplementary Figure S4, Figure 4). To examine further the similarities of the different complexes, we used principal component analysis (PCA) (Figure 4A). PCA plots for each segment of S7–S10 showed that the SHAPE profiles of the single transcripts were distanced from any multi-segmented complexes, consistent with the previous analysis. However, the SHAPE reactivity signals of the complete complex with mutant S10 were distinct from all other complexes (Figure 4A). Distribution analysis also showed that the addition of mutant S10 caused significant changes in the inter quartile ranges (Figure 4B). This data suggests that BTV RNA assembly is dynamic and that a change in a critical region of one key segment can significantly impact the contacts among all other segments in the complex.

### BTV RNA–RNA interactions and formation of complexes follow the similar sequential order in the host cells.

**Figure 5.**
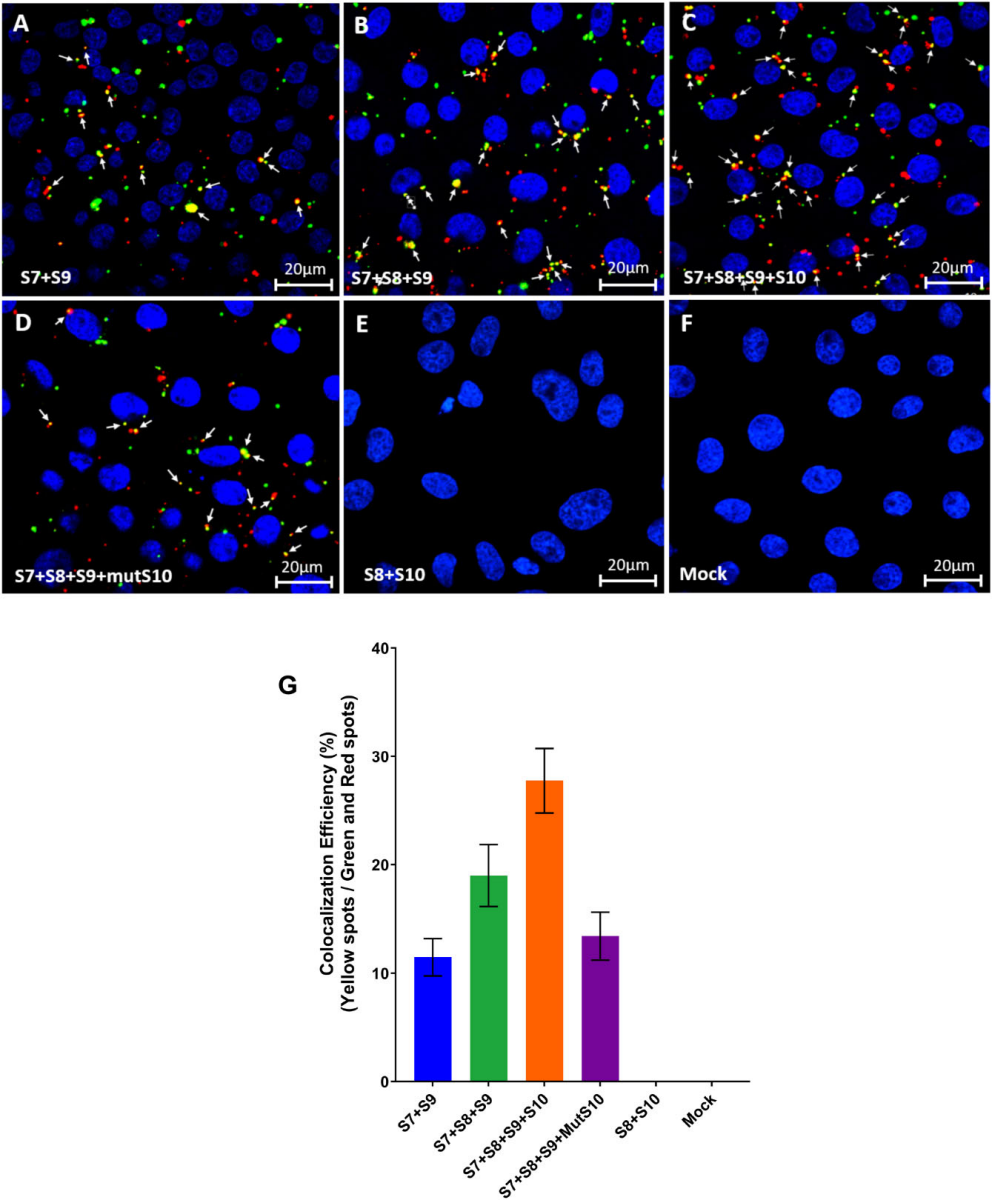
Viral ssRNAs fluorescence *in-situ* hybridization chain reaction (HCR) and colocalization analyses. *In-situ* HCR carried out in BSR cell monolayer fixed using multiple hairpin nucleotide probes. Two colour fluorophores were conjugated to the hairpin nucleotide probes: the Atto488 (496 nm, green) and Cy5 (670 nm, far-red) are independent detectors for the viral + ssRNA S7 and S9. (**A**) S7 + S9, (**B**) S7 + S8 + S9, (**C**) S7 + S8 + S9 + S10, (**D**) S7 + S8 + S9 + mutant S10 (mutS10), (**E**) S8 + S10 as negative control and (**F**) no RNA transfected (Mock). (**G**) The colocalization efficiency is calculated by dividing the number of colocalized spots (yellow) by the number of uncolocalised spots (green and red). The yellow spots show the colocalization between Atto488 and Cy5 spots, which are indicated by white arrows. A total of 15 images from different fields containing more than 150 cells were analyzed for each experiment. The mean standard error bars are shown.

To determine if BTV ssRNA segments form similar RNA complexes in the host cell cytoplasm, we next employed *in-situ* hybridisation chain reaction (HCR). We used 30 targeting probes whose binding to each RNA segment was first tested by using linearized fluorophore-labelled oligonucleotides (Supplementary Table S2, Supplementary Figure S5). First, segments S7 and S9 were labelled with Atto488 (green) and Cy5 (far red) fluorophores respectively. Then BSR cells were transfected with different RNA segment combinations, either labelled S7 + labelled S9 only (Figure 5A), or in presence of unlabelled S8 (Figure 5B), or in presence of unlabelled S8 + unlabelled S10 (Figure 5C). To avoid expression of BTV RNA binding proteins NS2 and VP6, which might influence any RNA–RNA interactions, the start codons (ATG) of S8 and S9 were changed to GTG, preventing translation while not impacting the nucleotide structure significantly.

Although S7 and S9 could co-localize when expressed together, colocalization was much more obvious in the presence of S8 (∼18%) and strongest (∼32%) when all four segments were transfected in a mixture. Further, when we used the S10 nts21–41 mutant RNA instead of wild-type S10 RNA (Figure 5D), the complex formation ratio dropped considerably (∼13%) (Figure 5G). To establish the specificity of the HCR, we used two negative controls, one with no fluorophore-labelled S8 and S10 transcripts (Figure 5E) and the other with no transcript (Figure 5F), both of which showed no signals of hybridization/RNA complex formation.

These data indicate that S7-S10 transcripts form complexes in the cytoplasm of cells similar to those observed in the *in vitro* assays described. The HCR data is not only consistent with the SHAPE data described, but also the *in vitro* data previously published (15).

### Determination of importance of the structural changes of RNA complexes in virus replication

SHAPE-MaP data demonstrated that a considerable number of RNA base-pairing interactions changed during complex formation. More importantly, many of these changes were observed at consecutive nucleotides, which could potentially form RNA binding motifs. A number of these motifs overlap or are in juxtaposition to the putative *trans*-acting sites previously predicted by computer modelling (15) (Table 1, Supplementary Table S3). To investigate their biological importance, we selected 13 sites among the smaller segments (S7–S10), each of which showed significant changes of SHAPE signals during complex formation. These sites, which were between 7 and 18 nucleotides in length based on the previous computer analysis (15), were then subjected to mutagenesis and their impact on virus recovery re-assessed by reverse genetics (RG). BTV RG has been successfully used previously to determine the functional effects of mutations in virus replication (31), showing that, for example, silent mutations (i.e. change of nucleotides but not amino acids), have a lethal effect on virus replication, consistent with a role for nucleotide sequences in genome packaging (15,17,39).

**Table 1.** Virus recovery of mutations (shown in bold) at S6-S10 RNA possible interacting sites identified by the ΔSHAPE

ΔSHAPE dataset	Segment	Sequence (wild type and mutated)	Matching computer prediction	Virus recovery
**Single vs S7–S9**	**S7**	[148–156] 5’ -AACUUUACGAG- 3’ **G** AC **AC** U **GA** G **G** G	**No**	**No**
Single vs S7–S9	**S7**	[377–388] 5’ -ACCAGCGCGUCA- 3’ **U** CC **G** GC **UA** G **A** CA	**Yes**	**No**
**Single vs S7–S9**	**S7**	[478–489] 5’-GUGGUCCGGAUA-3’ G **C** GG **G** CC **A** GA **C** A	**Yes**	**No**
**Single vs S7–S9 & S7–S9 vs S7–S10**	**S7**	[974–985] 5’-UUAUACUGUUUUA-3’ **A** UA **C** AC **A** GU **AC** U **C**	**No**	**No**
**Single vs S7–S9**	**S7 (3’ UTR)**	[1119–1133] 5’-UAUGUGACCCAUUCA- 3’ UA **C** GU **U** AC **U** CA **CAGU**	**Yes**	**No**
**S7–S10 vs S6–S10**	**S7 (3’ UTR)**	[1102–1116] 5’-GUGUCGGUUGUGGGA-3’ **ACACAAAAAACCC** GA	**No**	**No**
**Single vs S7–S9**	**S8**	[85–98] 5’-AUGCGAGAGCGAUCG-3’ **G** UG **U** G **G** G **GCAAU** U **GC**	**Yes**	**No**
**Single vs S7–S9**	**S8**	[168–180] 5’-AUCCGGAACCUAA-3’ A **C** CC **U** GA **G** CC **G** AA	**Yes**	**No**
**Single vs S7–S9**	**S8**	[343–353] 5’-ACAACAUAAUG-3’ **U** CA **G** CA **C** AA **C** G	**No**	**Attenuated**
**Single vs S7–S9**	**S8**	[378–390] 5’-AGUAUUGUAAAGG-3’ A **A** UA **C** UG **C** AA **G** GG	**No**	**Attenuated**
**Single vs S7–S9**	**S9**	[274–281] 5’-AGUGGGAUC-3’ **C** GU **A** GG **CAG**	**No**	**No**
**Single vs S7–S10 & S7–S10 vs S6–S10**	**S10**	[374–392] 5’-UAUAAUACAUAUGACAUUAC-3’ **G** AU **C** AU **C** CA **C** AUGAC **GC** U **UU**	**No**	**Attenuated**
**S7–S10 vs S6–S10**	**S10 (3’ UTR)**	[748–761] 5’-GUAGAGUGGUUGAU-3’ GUAG **CAACCAACG** U	**No**	**No**
**Single vs S6–S10**	**S6**	[296–310] 5’-AGCUUUUCCCAACCA-3’ A **U** CUUU **CAG** C **C** A **G** CC	**Yes**	**Yes**
**Single vs S6–S10**	**S6**	[1123–1136] 5’-GUGUUCGCCAUUCA-3’ **A** U **CAGUC** CC **U** UU **A** A	**Yes**	**Yes**
**Single vs S6–S10**	**S6**	[1281–1295] 5’-UUCACAUUCACCGCU-3’ U **C** CA **U** AU **A** CA **UA** G **A** U	**Yes**	**No**

**Figure 6.**
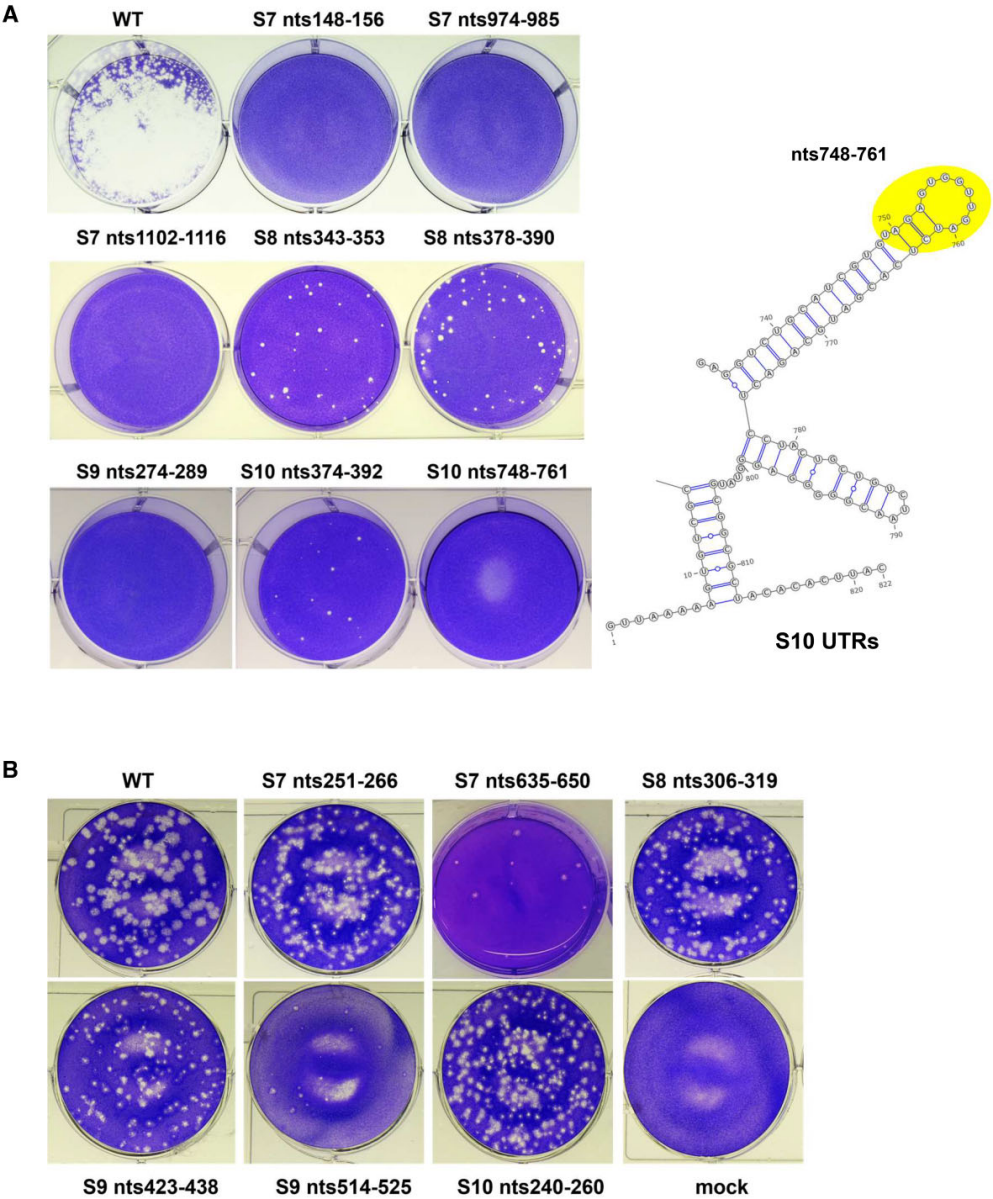
The impact of mutating the SHAPE identified RNA interacting sites on virus recovery. (**A**) Mutations were introduced to the indicated RNA interacting sites and virus recovery was performed. Plaques of the virus (if recovered) carrying the mutation are shown. As an example, the location of S10 nts748-761 is indicated in 5’ and 3’UTR of S10 secondary structure. (**B**) Mutations were introduced to the indicated sites with no SHAPE signal changes between different stages. Plaques of the virus (if recovered) carrying the mutation are shown.

Of the 13 selected sites for mutagenesis, six sites were targeted in S7, four in S8, one in S9 and two in S10. When silent mutations were introduced into these sites, the majority failed to recover virus, indicating they are critical for replication (Table 1, Supplementary Figure S2). However, two S8 mutations could recover virus although both were attenuated (Figure 6A). For the two sites in S10, one (nts748-761) hindered virus recovery while the other generated attenuated virus. It is noteworthy that this region of S10 is located in the long 3’-UTR of S10, which has previously been shown to be critical for BTV genome packaging and virus replication (17,26). Separately, we also introduced three mutations into the larger S6 segment, of which only one influenced virus growth (Table 1; Supplementary Figure S6).

Sites that remained comparatively unchanged during complex formation were also examined for their importance for virus replication. Regions identified with lower ΔSHAPE signals during Stage 1 and Stage 2 (standard deviation < 0.2, Supplementary Figure S2 and supplementary data) were mostly located in loop structures which may be part of intra-molecule interactions that did not change in the presence of other RNAs. Of these regions, six were selected in S7–S10 to represent non-reactive sequences, two from S7 (nts251–266; nts635–650), one from S8 (nts306–319), two from S9 (nts423–438; nts514–525) and one from S10 (nts240–260). All six sites were subjected to mutagenesis introducing silent mutations to alter the nucleotide sequences without affecting the reading frame. All six mutations still recovered viruses although in two cases, S7 nts635-650 and S9 nts514-525, smaller plaques were produced. (Table 2, Figure 6B). Overall, regions with lower ΔSHAPE values were less important for virus viability than regions with high ΔSHAPE values, consistent with a correlation between the ΔSHAPE value and an essential viral function, such as genome packaging.

**Table 2.** Virus recovery of mutations (shown in bold) in S7–S10 sites with no SHAPE changes in S7–S10

ΔSHAPE dataset	Segment	Sequence (wild type and mutated)	Virus recovery
**Single vs S7–S9**	**S7**	[251–266] 5’-ACCGAUAUCUCCAGAU-3’ **U** CC **A** AU **CAGC** CC **U** GA **C**	**Yes**
**Single vs S7–S9**	**S7**	[635–650] 5’-AGGCGUGACUGUUAGC-3’ **U** GG **A** GU **A** AC **C** GU **CUCU**	**Attenuated**
**Single vs S7–S9**	**S8**	[306–319] 5’-UUGAGGGUGUCAGUG-3’ U **C** GA **A** GG **A** GU **GUCA** G	**Yes**
**Single vs S7–S9**	**S9**	[423–438] 5’-AGAAGAGAUUGCUCGC-3’ AG **G** A **A** A **U** A **GCAAGA** GC	**Yes**
**Single vs S7–S9**	**S9**	[514–525] 5’-CGCAGUCUCCAG-3’ **A** G **AUCA** CU **A** CA **A**	**Attenuated**
**Single vs S7–S10**	**S10**	[240–260] 5’-AGAAGGCUGCAUUCGCAUCGU-3’ A **A** AA **A** GC **A** GC **C** UUUGC **UAGU** U	**Yes**

**Figure 7.**
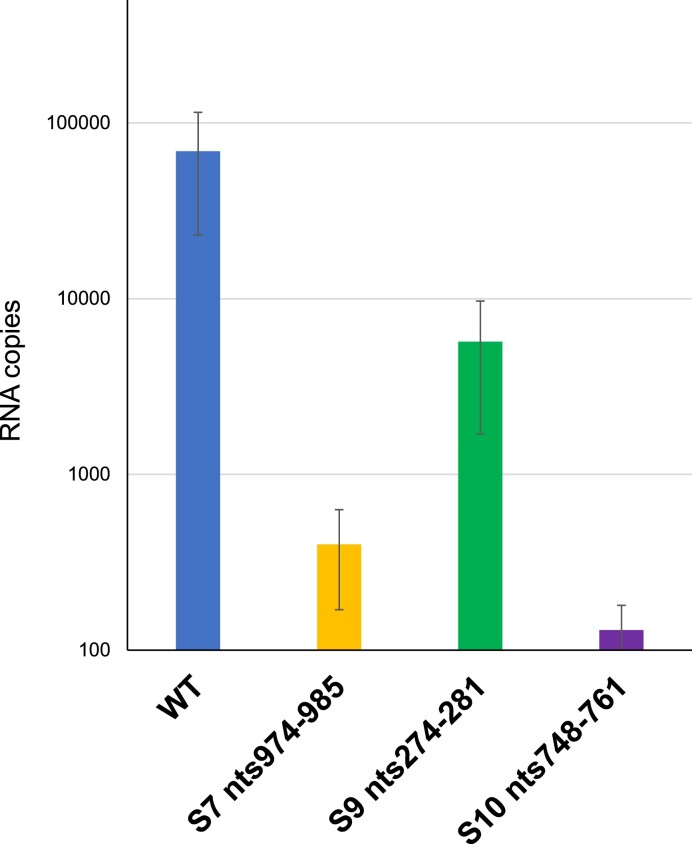
The impact of mutating the SHAPE identified RNA interacting sites for *in vitro* RNA packaging efficiency. Mutant segments as indicated were used in the *in vitro* CFA assay. The packaged RNA was quantified and the result of three individual experiments are shown. The mean standard error bars are shown.

To further confirm that the failure of virus recovery associated with high ΔSHAPE signals was due to genome packaging, we utilised an *in vitro* RNA packaging assay to measure how the identified mutations influenced ten RNA segment packaging (14,17). Three sites (S7 nts974–985, S9 nts274–281 and S10 nts748–761), identified by SHAPE and shown to be critical for virus recovery, were tested using the previously established *in vitro* transcription/translation-based cell-free assembly (CFA) assay as described in Methods. The RNA packaged into protein capsids was measured by qRT-PCR (Figure 7). Mutation of S9 nts274–281 led to a 10-fold decrease in RNA packaging; mutation of S7 nts974–985 a 100-fold decrease, and mutation of S10 nts748–761 a greater than 500-fold decrease. These data confirm that the disruption of RNA interacting sites, which interferes with RNA complex formation, also leads to failed RNA packaging, consistent with the suggestion that the RNA:RNA interaction mapped in this work is a necessity for physical incorporation of RNA into assembling capsids.

## Discussion

How RNA viruses with segmented genomes, correctly package their complete genomes has been a long-standing question. One attractive hypothesis is that the single stranded form of the genomic segments utilize specific RNA–RNA interactions to form a network for assembly rather than rely on a sequential packaging of each segment into the newly assembling capsid (15,17,25,26). The paradigm is influenza, the current model of which is that a network of sequence specific interactions among the viral ribonucleoproteins results in a supramolecular complex that is incorporated directly into budding viral particles (11,29,40–42). For BTV and other related viruses, the mechanism is likely to differ as packaging can be initiated by a single segment (17) and no segment is complexed with a ribonucleoprotein. Previous EMSA data have suggested that the smaller segments (S7–S10) of BTV form complexes that are critical for genome packaging (15,26).

In this study, we provide the first direct evidence of how these smaller segments participate in the RNA assembly of the ten segments of the BTV genome. We discovered that large scale rearrangements among the four smallest segments (S7–S10) of BTV are essential to the establishment of a robust core RNA complex, with each of the four segments undergoing significant changes prior to the sequential recruitment of the remaining larger RNA segments. The selection of the four smaller segments also enhanced their interactions with each other in the absence of larger segments. While incoming larger segments still altered the core complex, the scale of the changes was significantly lower than the changes which occurred during core formation. This scenario is evident for all smaller segments, particularly during the formation of the S7-S9 complex, which had the largest impact on SHAPE signals. Additionally, S10 alone significantly altered the SHAPE signals when it was added to the S7–S9 complex. In contrast, when the larger S6 was added to the S7–S10 complex, the impact on each of the four smaller segments was insignificant. The data confirm our earlier findings that an essential component of the BTV genome complex is a core formed by the four smaller segments. We confirmed biological relevance by showing that mutations introduced into the interacting sites present in the smaller segments were largely not tolerated for virus growth whereas changes in the larger segments, such as S6, allowed virus recovery in most cases. These *in vitro* data were further substantiated by *in vivo in-situ* hybridization data, which confirmed that complexes of S7–S9 and S7–S10, form in the cytoplasm of transfected cells. This data confirms that such interactions are independent of any viral protein, re-enforcing their mechanistic distinction from the virus models which use virally encoded proteins to sort and package the genome (3,8,12,43).

When comparing the SHAPE data obtained here with the previously predicted interacting sites (15), most predicted sites either coincided with, or were in the vicinity of, the regions identified by SHAPE-MaP. Interestingly, certain predicted interaction sites in the S7–S9 complex, were identified by the SHAPE data only when S10 was present, stressing again the unique contribution made by S10 to the S7–S10 complex. Being shorter, the S7–S10 transcripts will emerge sooner from the transcriptional active core following infection, possibly relating to our observed distinction between Stage 1 and Stage 2 complexes. How the choice is made between engaging a ribosome for translation or entering an RNA:RNA complex for packaging is not known but it is likely to involve concentration and cellular environment, notably the formation of the liquid-liquid phase separation (LLPS) structures by viral encoded NS2 (44). Alternately, Stage 1 and 2, which were based on energy calculations in our previous study (15), may not reflect the cellular situation.

The SHAPE-MaP profiles revealed both decreases and increases in reactivities during complex formation, which is suggestive of global RNA structural rearrangements. Our attempt to virus recovery demonstrated that both annealing (RNA–RNA) and releasing (of RNA–RNA interaction) can be functionally important, as in both scenarios the interaction sites were shown to be critical. It was evident from the changes during RNA complex expansion that some regions were involved in multiple steps in the process. For example, S8 nts410–430 had its SHAPE signal increased at Stage 1, whilst the same region had a decreased signal at Stage 2. Similarly, S8 nts520–540 had a decreased signal at Stage 2, but at Stage 3, it had an increased peak. S10 nts740–760, a region in the 3’-UTR previously shown to be critical (26), had significant changes at every stage, from single segment addition to S7–S10, to S6–S10, to S3–S10 and the complete S1–S10. This suggests that it is less the formation a stable secondary structure that is important for packaging but the adoption of the flexibility that allows the recognition of other segments. Consistent with this, the recent SHAPE-MaP analysis of rotavirus, a member of the *Reoviridae* virus with 11 genome segments has shown that rotavirus non-structural NSP2, a chaperone protein (equivalent to BTV NS2), did not significantly change the SHAPE profile of a mixture of 11 genome transcripts but increased their flexibility to allow interaction (45). This data is consistent with the data above, that the secondary structures of RNA are not significantly altered by viral proteins, unless or until the formation of the LLPS structures that sequester them for assembly (44,46).

How viral proteins recognize correctly collated ssRNAs complexes remains unclear, but a role for the smallest BTV structural protein, VP6, and the inner capsid protein VP3 has been reported (47). Further, we recently confirmed a role for VP6 in packaging BTV S10 ssRNA into the assembling VP3 capsid (48). Although further studies will be required to define the finer details of the RNA packaging process of BTV, the data we presented in this study, elucidates a vital stage of the Orbivirus genome assembly process and provides key information for the studying of other viruses with segmented RNA genome.

## Supplementary Material

gkae404_Supplemental_File

## Data Availability

The sequencing data have been deposited with the NCBI SRA under BioProject ID PRJEB71404. (https://www.ebi.ac.uk/ena/browser/view/PRJEB71404). The SHAPE analysis codes and TSV files produced by ΔSHAPE are available at https://doi.org/10.6084/m9.figshare.25713279.v1.

## References

[1] Adams R.L. , Pirakitikulr N., Pyle A.M. Functional RNA structures throughout the hepatitis C virus genome. Curr. Opin. Virol. 2017; 24:79–86. 28511116 10.1016/j.coviro.2017.04.007 PMC5529263

[2] Gaucherand L. , Iyer A., Gilabert I., Rycroft C.H., Gaglia M.M. Cut site preference allows influenza A virus PA-X to discriminate between host and viral mRNAs. Nat. Microbiol. 2023; 8:1304–1317. 37349586 10.1038/s41564-023-01409-8 PMC10690756

[3] Masters P.S. Coronavirus genomic RNA packaging. Virology. 2019; 537:198–207. 31505321 10.1016/j.virol.2019.08.031 PMC7112113

[4] Patel N. , White S.J., Thompson R.F., Bingham R., Weiss E.U., Maskell D.P., Zlotnick A., Dykeman E., Tuma R., Twarock R. et al. HBV RNA pre-genome encodes specific motifs that mediate interactions with the viral core protein that promote nucleocapsid assembly. Nat. Microbiol. 2017; 2:17098. 28628133 10.1038/nmicrobiol.2017.98 PMC5495169

[5] Patel N. , Wroblewski E., Leonov G., Phillips S.E.V., Tuma R., Twarock R., Stockley P.G. Rewriting nature's assembly manual for a ssRNA virus. Proc. Natl. Acad. Sci. U.S.A. 2017; 114:12255–12260. 29087310 10.1073/pnas.1706951114 PMC5699041

[6] Twarock R. , Towers G.J., Stockley P.G. Molecular frustration: a hypothesis for regulation of viral infections. Trends Microbiol. 2023; 32:17–26. 37507296 10.1016/j.tim.2023.07.003

[7] Ye L. , Ambi U.B., Olguin-Nava M., Gribling-Burrer A.S., Ahmad S., Bohn P., Weber M.M., Smyth R.P. RNA structures and their role in selective genome packaging. Viruses. 2021; 13:1788. 34578369 10.3390/v13091788 PMC8472981

[8] Ding P. , Kharytonchyk S., Waller A., Mbaekwe U., Basappa S., Kuo N., Frank H.M., Quasney C., Kidane A., Swanson C. et al. Identification of the initial nucleocapsid recognition element in the HIV-1 RNA packaging signal. Proc. Natl. Acad. Sci. U.S.A. 2020; 117:17737–17746. 32647061 10.1073/pnas.2008519117 PMC7395439

[9] Chou Y.Y. , Vafabakhsh R., Doganay S., Gao Q., Ha T., Palese P. One influenza virus particle packages eight unique viral RNAs as shown by FISH analysis. Proc. Natl. Acad. Sci. U.S.A. 2012; 109:9101–9106. 22547828 10.1073/pnas.1206069109 PMC3384215

[10] Jakob C. , Paul-Stansilaus R., Schwemmle M., Marquet R., Bolte H. The influenza A virus genome packaging network - complex, flexible and yet unsolved. Nucleic Acids Res. 2022; 50:9023–9038. 35993811 10.1093/nar/gkac688 PMC9458418

[11] Li X. , Gu M., Zheng Q., Gao R., Liu X. Packaging signal of influenza A virus. Virol. J. 2021; 18:36. 33596956 10.1186/s12985-021-01504-4 PMC7890907

[12] Noda T. , Murakami S., Nakatsu S., Imai H., Muramoto Y., Shindo K., Sagara H., Kawaoka Y. Importance of the 1+7 configuration of ribonucleoprotein complexes for influenza A virus genome packaging. Nat. Commun. 2018; 9:54. 29302061 10.1038/s41467-017-02517-w PMC5754346

[13] Chen D. , Patton J.T. De novo synthesis of minus strand RNA by the rotavirus RNA polymerase in a cell-free system involves a novel mechanism of initiation. RNA. 2000; 6:1455–1467. 11073221 10.1017/s1355838200001187 PMC1370016

[14] Lourenco S. , Roy P. In vitro reconstitution of Bluetongue virus infectious cores. Proc. Natl. Acad. Sci. U.S.A. 2011; 108:13746–13751. 21808007 10.1073/pnas.1108667108 PMC3158217

[15] AlShaikhahmed K. , Leonov G., Sung P.Y., Bingham R.J., Twarock R., Roy P. Dynamic network approach for the modelling of genomic sub-complexes in multi-segmented viruses. Nucleic Acids Res. 2018; 46:12087–12098. 30299495 10.1093/nar/gky881 PMC6294558

[16] Burkhardt C. , Sung P.Y., Celma C.C., Roy P. Structural constraints in the packaging of bluetongue virus genomic segments. J. Gen. Virol. 2014; 95:2240–2250. 24980574 10.1099/vir.0.066647-0 PMC4165931

[17] Sung P.Y. , Roy P. Sequential packaging of RNA genomic segments during the assembly of Bluetongue virus. Nucleic Acids Res. 2014; 42:13824–13838. 25428366 10.1093/nar/gku1171 PMC4267631

[18] Roy P. Knipe D.M. , Howley P.M. Fields' Virology. 2007; 6th edn. USA Lippincott Williams & Wilkins 1975–1997.

[19] Fukusho A. , Yu Y., Yamaguchi S., Roy P. Completion of the sequence of bluetongue virus serotype 10 by the characterization of a structural protein, VP6, and a non-structural protein, NS2. J. Gen. Virol. 1989; 70:1677–1689. 2544660 10.1099/0022-1317-70-7-1677

[20] Lawton J.A. , Estes M.K., Prasad B.V. Three-dimensional visualization of mRNA release from actively transcribing rotavirus particles. Nat. Struct. Biol. 1997; 4:118–121. 9033591 10.1038/nsb0297-118

[21] He Y. , Shivakoti S., Ding K., Cui Y., Roy P., Zhou Z.H. In situ structures of RNA-dependent RNA polymerase inside bluetongue virus before and after uncoating. Proc. Natl. Acad. Sci. U.S.A. 2019; 116:16535–16540. 31350350 10.1073/pnas.1905849116 PMC6697807

[22] Periz J. , Celma C., Jing B., Pinkney J.N., Roy P., Kapanidis A.N. Rotavirus mRNAS are released by transcript-specific channels in the double-layered viral capsid. Proc. Natl. Acad. Sci. U.S.A. 2013; 110:12042–12047. 23818620 10.1073/pnas.1220345110 PMC3718169

[23] Van Dijk A.A. , Huismans H. In vitro transcription and translation of bluetongue virus mRNA. J. Gen. Virol. 1988; 69:573–581. 2832524 10.1099/0022-1317-69-3-573

[24] Roy P. Bluetongue virus structure and assembly. Curr. Opin. Virol. 2017; 24:115–123. 28609677 10.1016/j.coviro.2017.05.003

[25] Fajardo T. , Sung P.Y., Celma C.C., Roy P. Rotavirus genomic RNA complex forms via specific RNA–RNA interactions: disruption of RNA complex inhibits virus infectivity. Viruses. 2017; 9:167. 28661470 10.3390/v9070167 PMC5537659

[26] Fajardo T. Jr , Sung P.Y., Roy P Disruption of specific RNA–RNA interactions in a double-stranded RNA virus inhibits genome packaging and virus infectivity. PLoS Pathog. 2015; 11:e1005321. 26646790 10.1371/journal.ppat.1005321 PMC4672896

[27] Boerneke M.A. , Ehrhardt J.E., Weeks K.M. Physical and functional analysis of viral RNA genomes by SHAPE. Annu Rev Virol. 2019; 6:93–117. 31337286 10.1146/annurev-virology-092917-043315 PMC6768749

[28] Busan S. , Weidmann C.A., Sengupta A., Weeks K.M. Guidelines for SHAPE reagent choice and detection strategy for RNA structure probing studies. Biochemistry. 2019; 58:2655–2664. 31117385 10.1021/acs.biochem.8b01218 PMC6712974

[29] Dadonaite B. , Gilbertson B., Knight M.L., Trifkovic S., Rockman S., Laederach A., Brown L.E., Fodor E., Bauer D.L.V. The structure of the influenza A virus genome. Nat. Microbiol. 2019; 4:1781–1789. 31332385 10.1038/s41564-019-0513-7 PMC7191640

[30] Smola M.J. , Rice G.M., Busan S., Siegfried N.A., Weeks K.M. Selective 2'-hydroxyl acylation analyzed by primer extension and mutational profiling (SHAPE-MaP) for direct, versatile and accurate RNA structure analysis. Nat. Protoc. 2015; 10:1643–1669. 26426499 10.1038/nprot.2015.103 PMC4900152

[31] Boyce M. , Celma C.C., Roy P. Development of reverse genetics systems for bluetongue virus: recovery of infectious virus from synthetic RNA transcripts. J. Virol. 2008; 82:8339–8348. 18562540 10.1128/JVI.00808-08 PMC2519640

[32] Madden E.A. , Plante K.S., Morrison C.R., Kutchko K.M., Sanders W., Long K.M., Taft-Benz S., Cruz Cisneros M.C., White A.M., Sarkar S. et al. Using SHAPE-MaP to model RNA secondary structure and identify 3'UTR variation in chikungunya virus. J. Virol. 2020; 94:e00701-20. 32999019 10.1128/JVI.00701-20 PMC7925192

[33] Incarnato D. , Morandi E., Simon L.M., Oliviero S. RNA Framework: an all-in-one toolkit for the analysis of RNA structures and post-transcriptional modifications. Nucleic Acids Res. 2018; 46:e97. 29893890 10.1093/nar/gky486 PMC6144828

[34] Haimovich G. , Gerst J.E. Single-molecule fluorescence in situ hybridization (smFISH) for RNA detection in adherent animal cells. Bio Protoc. 2018; 8:e3070. 10.21769/BioProtoc.3070 PMC8342053 34532531

[35] Tsuneoka Y. , Funato H. Modified in situ hybridization chain reaction using short hairpin DNAs. Front. Mol. Neurosci. 2020; 13:75. 32477063 10.3389/fnmol.2020.00075 PMC7235299

[36] Dirks R.M. , Pierce N.A. Triggered amplification by hybridization chain reaction. Proc. Natl. Acad. Sci. U.S.A. 2004; 101:15275–15278. 15492210 10.1073/pnas.0407024101 PMC524468

[37] Matrosovich M. , Matrosovich T., Garten W., Klenk H.D. New low-viscosity overlay medium for viral plaque assays. Virol. J. 2006; 3:63. 16945126 10.1186/1743-422X-3-63 PMC1564390

[38] Busan S. , Weeks K.M. Accurate detection of chemical modifications in RNA by mutational profiling (MaP) with ShapeMapper 2. RNA. 2018; 24:143–148. 29114018 10.1261/rna.061945.117 PMC5769742

[39] Sung P.Y. , Zhou Y., Kao C.C., Aburigh A.A., Routh A., Roy P. A multidisciplinary approach to the identification of the protein-RNA connectome in double-stranded RNA virus capsids. Nucleic Acids Res. 2023; 51:5210–5227. 37070191 10.1093/nar/gkad274 PMC10250232

[40] Fournier E. , Moules V., Essere B., Paillart J.C., Sirbat J.D., Isel C., Cavalier A., Rolland J.P., Thomas D., Lina B. et al. A supramolecular assembly formed by influenza A virus genomic RNA segments. Nucleic Acids Res. 2012; 40:2197–2209. 22075989 10.1093/nar/gkr985 PMC3300030

[41] Piasecka J. , Lenartowicz E., Soszynska-Jozwiak M., Szutkowska B., Kierzek R., Kierzek E. RNA secondary structure motifs of the influenza A virus as targets for siRNA-mediated RNA interference. Mol. Ther. Nucleic Acids. 2020; 19:627–642. 31945726 10.1016/j.omtn.2019.12.018 PMC6965531

[42] Kesy J. , Patil K.M., Kumar S.R., Shu Z., Yong H.Y., Zimmermann L., Ong A.A.L., Toh D.K., Krishna M.S., Yang L. et al. A short chemically modified dsRNA-binding PNA (dbPNA) inhibits influenza viral replication by targeting viral RNA panhandle structure. Bioconjug. Chem. 2019; 30:931–943. 30721034 10.1021/acs.bioconjchem.9b00039

[43] Strauss S. , Acker J., Papa G., Desiro D., Schueder F., Borodavka A., Jungmann R. Principles of RNA recruitment to viral ribonucleoprotein condensates in a segmented dsRNA virus. eLife. 2023; 12:e68670. 36700549 10.7554/eLife.68670 PMC9925054

[44] Rahman S.K. , Ampah K.K., Roy P. Role of NS2 specific RNA binding and phosphorylation in liquid-liquid phase separation and virus assembly. Nucleic Acids Res. 2022; 50:11273–11284. 36259663 10.1093/nar/gkac904 PMC9638936

[45] Coria A. , Wienecke A., Knight M.L., Desiro D., Laederach A., Borodavka A. Rotavirus RNA chaperone mediates global transcriptome-wide increase in RNA backbone flexibility. Nucleic Acids Res. 2022; 50:10078–10092. 36062555 10.1093/nar/gkac738 PMC9508848

[46] Nichols S.L. , Nilsson E.M., Brown-Harding H., LaConte L.E.W., Acker J., Borodavka A., McDonald Esstman S. Flexibility of the rotavirus NSP2 C-terminal region supports factory formation via liquid-liquid phase separation. J. Virol. 2023; 97:e0003923. 36749077 10.1128/jvi.00039-23 PMC9973012

[47] Matsuo E. , Yamazaki K., Tsuruta H., Roy P. Interaction between a unique minor protein and a major capsid protein of bluetongue virus controls virus infectivity. J. Virol. 2018; 92:e01784-17. 29142128 10.1128/JVI.01784-17 PMC5774872

[48] Xia X. , Sung P.Y., Martynowycz M.W., Gonen T., Roy P., Zhou Z.H. RNA genome packaging and capsid assembly of bluetongue virus visualized in host cells. Cell. 2024; 187:2236–2249. 38614100 10.1016/j.cell.2024.03.007 PMC11182334

