# Supplementary material for: Recruitment of multi-segment genomic RNAs by Bluetongue virus requires a preformed RNA network
Source: Nucleic Acids Res. 2024 May 20;52(14):8500–14. doi: 10.1093/nar/gkae404 (PMC11317150; doi:10.1093/nar/gkae404)
Supplement: gkae404_Supplemental_File [file gkae404_supplemental_file.pdf]

Supplementary Table 1. Primers used for mutagenesis

Segment	Sequence	Primers
S7	[148-156]	F GGATTGACAC TGAGGGGGGT GACGATGCGC<br>R CACCCCCCTCAGTGTCAATCCATTATATCTG
S7	[377-388]	F GGTCCG GCTAGACAGC CCTATGGTTT TTTCCTTGAA ACTGAAG<br>R GCTGTCTAGCCGGACCCCACGTCGAAGTCTCCCCAGTCAC
S7	[478-489]	F GTGCG GGCCAGACAT GATTCAAGTG TCACTGAATG CTGGAGC<br>R CATGTCTGGCCCGCACACTACCGCAGTTACTGCTTGAGC
S7	[974-985]	F GATGTATACACA GTACTCAGACCTGAGTTCGCGATCCAC<br>R GTCTGAGTACTGTGTATACATCAGCAAGCGTAGAC
S7<br>(3'UTR)	[1102-1116]	F GTACACAAAAA ACCCGATATA TGTGACCC<br>R CGGGTTTTTTGTGTACCGCATATGTAACCCAC
S7<br>(3'UTR)	[1119-1133]	F TCGGTTGTGGGATATACGTTACTCACAGTAACGTCTCTTAGATTACAC<br>R CTAAGAGACGTTACTGTGAGTAACGTATATCCCACAACCGACACACCGC
S8	[85-98]	F TTGTGTG GGGCAATTGC AAAGTTGAGT TCGCAACCGT ATTGTC<br>R GCAATTGCCCCACACAATGTTTTGCCATTTGCATCCAAAAC
S8	[168-180]	F AAACCCTGAGCCGA AGGGATACGT GCTGAATGTT CCAGGACC<br>R CCTTCGGCTCAGGGTTTTTGACAGGTTTAAAAGCTATTACTC
S8	[343-353]	F GGTTCAGCACA ACGGAGTAAT GGTTGATGCTGAG<br>R ATTACTCCGTTGTGCTGAACCCTAGTAGCCATTG
S8	[378-390]	F GATCAAATACTGCAAGGG AATGGGAATA GTGC<br>R CCCTTGCAGTATTTGATCTCAGCATCAACC
S9	[274-281]	F GCCGTAGGCA GCGGATCAAG CGCAAAAG<br>R CCGCTGCCTACGGCAGTATGTATGCGTC
S10	[374-392]	F GCGATCATCCACATGACGCTTTTGATAGCG GCGGTTGTTG<br>R CAAAAGCGTCATGTGGATGATCGCTCTTTTCTTCTTTAAG
S10<br>(3' UTR)	[748-761]	F GTAGCAACCAACG TCTCACGATG CAGACTC<br>R GACGTTGGTTGCTACACGATGCAGACCTCG
S6	[296-310]	F TCCAATGCTTCAAATCTTTCAGCCAGCCGTACGAAGAGGATGTCGAAGGGAAGAT<br>R ATCCTCTTCGTACGGCTGGCTGAAAGATTTGAAGCATGGAGCCAGACTGTCTCCCGATCATAC
S6	[1123-1136]	F CTGGATGCAGT CCCTTTAAGA CGGTGAAGAT TGAG<br>R GTCTTAAAGGGACTGCATCCAGTTAGTTCTGATGCGC
S6	[1281-1295]	F GATCCATATACATAGATGGATTAGAGGCTGTGGCATCTGGAAC<br>R CATCTATGTATATGGATCGCAGTTGTAATCAAGGCCTCCGCCC
S7	[251-266]	F GGTCCAATCAGC CCTGACTATA CCCAACATAT GGCTAC<br>R ATAGTCAGGGCTGATTGGACCTACGTTTATCCCAG
S7	[635-650]	F GCTGGAGT AACCGTCTCT GTTGGTGGAG TAGATATG<br>R CAGAGACGGTTACTCCAGCTTGAGTTTGCTGTGAG
S8	[306-319]	F GTTCGAA GGAGTGTCAG TAACGCCAAT GGCTACTAG<br>R TACTGACACTCCTTCGAACTTCCACTCTTCCC
S9	[423-438]	F ACTGAGGAAA TAGCAAGAGC GATCGAATCT AAATAC<br>R GCTCTTGCTATTTCCTCAGTTAGAACTACCCATCTTC
S9	[514-525]	F GAAAGATCAC TACAAAAAGA GCTAGGGATT TCGCG<br>R CTTTTTGTAGTGATCTTTCTACTTCGATGATTTGTG
S10	[240-260]	F GAAAAAGCAGCC TTTGCTAGTT ACGCAGAAGC GTTTCGTG<br>R GTAACTAGCAAAGGCTGCTTTTTCCGCTTTTTGTGTTTG

Supplementary Table 2. RNA probes used for in situ hybridization

Probe #	Probe (5'-> 3')	Probe position *	Percent GC
BTV1-wt-S10_RNA-HCR-Probe-H1_5'-cy5	cy5 - 5' - TGTTGCAAAGGAACGTCGAGCTGTAATGGTGCTCGACGTTCC - 3'		
BTV1-wt-S10_RNA-HCR-Probe-H2_3'-cy5	5' - GCTCGACGTTCCTTTGCAACAGGAACGTCGAGCACCATTACA - 3' - cy5		
BTV1-WT-S9_RNA-HCR-target_1	gcagctgacatatgcgatAAAGCTCGACGTTCCTTTGCAACA	9	50.00%
BTV1-WT-S9_RNA-HCR-target_2	cgtcaccgggtgcaagaaAAAGCTCGACGTTCCTTTGCAACA	32	61.00%
BTV1-WT-S9_RNA-HCR-target_3	ctcctcggatgaacgcttAAAGCTCGACGTTCCTTTGCAACA	55	56.00%
BTV1-WT-S9_RNA-HCR-target_4	atttggatctgtctctgtAAAGCTCGACGTTCCTTTGCAACA	78	39.00%
BTV1-WT-S9_RNA-HCR-target_5	gtctatccacatctgcatAAAGCTCGACGTTCCTTTGCAACA	242	44.00%
BTV1-WT-S9_RNA-HCR-target_6	ggatcccactgcagtatgAAAGCTCGACGTTCCTTTGCAACA	265	56.00%
BTV1-WT-S9_RNA-HCR-target_7	gttctccaggtcctttgAAAGCTCGACGTTCCTTTGCAACA	293	50.00%
BTV1-WT-S9_RNA-HCR-target_8	ttccatctcctctgtcaaAAAGCTCGACGTTCCTTTGCAACA	326	44.00%
BTV1-WT-S9_RNA-HCR-target_9	ctccaactcccgcatcagAAAGCTCGACGTTCCTTTGCAACA	368	61.00%
BTV1-WT-S9_RNA-HCR-target_10	atcttcctccattcgctcAAAGCTCGACGTTCCTTTGCAACA	392	50.00%
BTV1-WT-S9_RNA-HCR-target_11	catcaatcttcgtaccgtAAAGCTCGACGTTCCTTTGCAACA	455	44.00%
BTV1-WT-S9_RNA-HCR-target_12	tgatttgtgctgggacctAAAGCTCGACGTTCCTTTGCAACA	485	50.00%
BTV1-WT-S9_RNA-HCR-target_13	gctctttctggagactgcAAAGCTCGACGTTCCTTTGCAACA	515	56.00%
BTV1-WT-S9_RNA-HCR-target_14	cactccctcacgcgaaatAAAGCTCGACGTTCCTTTGCAACA	538	56.00%
BTV1-WT-S9_RNA-HCR-target_15	ctcttctcttttcgtcgcAAAGCTCGACGTTCCTTTGCAACA	585	50.00%
BTV1-WT-S9_RNA-HCR-target_16	cttcttcccttgtttacgAAAGCTCGACGTTCCTTTGCAACA	637	44.00%
BTV1-WT-S9_RNA-HCR-target_17	tctctctgcgcatcaccaAAAGCTCGACGTTCCTTTGCAACA	660	56.00%
BTV1-WT-S9_RNA-HCR-target_18	tcttcggacgtcttctctAAAGCTCGACGTTCCTTTGCAACA	690	50.00%
BTV1-WT-S9_RNA-HCR-target_19	tagtgatcccgacactcgAAAGCTCGACGTTCCTTTGCAACA	713	56.00%
BTV1-WT-S9_RNA-HCR-target_20	tcttctgactcatgacccAAAGCTCGACGTTCCTTTGCAACA	737	50.00%
BTV1-WT-S9_RNA-HCR-target_21	accgccaatcatgctcaaAAAGCTCGACGTTCCTTTGCAACA	760	50.00%
BTV1-WT-S9_RNA-HCR-target_22	cctattggagccatctttAAAGCTCGACGTTCCTTTGCAACA	786	44.00%
BTV1-WT-S9_RNA-HCR-target_23	acataactgcgctctcccAAAGCTCGACGTTCCTTTGCAACA	809	56.00%
BTV1-WT-S9_RNA-HCR-target_24	tagctcgcaccacatcttAAAGCTCGACGTTCCTTTGCAACA	845	50.00%
BTV1-WT-S9_RNA-HCR-target_25	cggttggcgctgtgaaatAAAGCTCGACGTTCCTTTGCAACA	869	56.00%
BTV1-WT-S9_RNA-HCR-target_26	cacctccttccaatgtggAAAGCTCGACGTTCCTTTGCAACA	892	56.00%
BTV1-WT-S9_RNA-HCR-target_27	ccgtactcgtgtatgctaAAAGCTCGACGTTCCTTTGCAACA	941	50.00%
BTV1-WT-S9_RNA-HCR-target_28	gcaggaactccgttttcaAAAGCTCGACGTTCCTTTGCAACA	968	50.00%
BTV1-WT-S9_RNA-HCR-target_29	tggaccctttagaggtgaAAAGCTCGACGTTCCTTTGCAACA	996	50.00%
BTV1-WT-S9_RNA-HCR-target_30	cgccctacgtcaagaaggAAAGCTCGACGTTCCTTTGCAACA	1019	61.00%

Probe #	Probe (5'-> 3')	Probe position *	Percent GC
BTV1-wt-S7_RNA-HCR-Probe-H1_5'-Atto488	Atto488 - 5' - TAGACTGAACCCACTCCGACGATCTGTCTTCGTCGGAGTGGG - 3'		
BTV1-wt-S7_RNA-HCR-Probe-H2_3'-Atto488	5' - CGTCGGAGTGGGTTCAGTCTACCCACTCCGACGAAGACAGAT - 3' - Atto488		
BTV1-WT-S7_RNA-HCR-target_1	cgatagtgtccatctctataAAACGTCGGAGTGGGTTCAGTCTA	11	40.00%
BTV1-WT-S7_RNA-HCR-target_2	atcacagtaagtgctcttgcAAACGTCGGAGTGGGTTCAGTCTA	33	45.00%
BTV1-WT-S7_RNA-HCR-target_3	ttgaagcgtagcacatgctcAAACGTCGGAGTGGGTTCAGTCTA	55	50.00%
BTV1-WT-S7_RNA-HCR-target_4	cagtatctccatcacgttagAAACGTCGGAGTGGGTTCAGTCTA	97	45.00%
BTV1-WT-S7_RNA-HCR-target_5	aacatctcatttctctgcgcAAACGTCGGAGTGGGTTCAGTCTA	183	45.00%
BTV1-WT-S7_RNA-HCR-target_6	gcggacagcatcatatctaaAAACGTCGGAGTGGGTTCAGTCTA	213	45.00%
BTV1-WT-S7_RNA-HCR-target_7	agatatcggtcctacgtttaAAACGTCGGAGTGGGTTCAGTCTA	241	40.00%
BTV1-WT-S7_RNA-HCR-target_8	gccatatgttgggtataatcAAACGTCGGAGTGGGTTCAGTCTA	264	40.00%
BTV1-WT-S7_RNA-HCR-target_9	cgtcgctagtacaccaattgAAACGTCGGAGTGGGTTCAGTCTA	286	50.00%
BTV1-WT-S7_RNA-HCR-target_10	cgcttccgttgtaaaaggtaAAACGTCGGAGTGGGTTCAGTCTA	313	45.00%
BTV1-WT-S7_RNA-HCR-target_11	tcacgcgagcaatctcattcAAACGTCGGAGTGGGTTCAGTCTA	335	50.00%
BTV1-WT-S7_RNA-HCR-target_12	aaaaaccatagggctgacgcAAACGTCGGAGTGGGTTCAGTCTA	383	50.00%
BTV1-WT-S7_RNA-HCR-target_13	ggttggaaagtctcttcagtAAACGTCGGAGTGGGTTCAGTCTA	411	45.00%
BTV1-WT-S7_RNA-HCR-target_14	ttgaatcatatccggaccacAAACGTCGGAGTGGGTTCAGTCTA	478	45.00%
BTV1-WT-S7_RNA-HCR-target_15	cctgaaatatctgctgcacaAAACGTCGGAGTGGGTTCAGTCTA	527	45.00%
BTV1-WT-S7_RNA-HCR-target_16	tatcatcatggggtcgttacAAACGTCGGAGTGGGTTCAGTCTA	550	45.00%
BTV1-WT-S7_RNA-HCR-target_17	ccatcgcgaagttttcaattAAACGTCGGAGTGGGTTCAGTCTA	587	40.00%
BTV1-WT-S7_RNA-HCR-target_18	tactccaccaacgctaacagAAACGTCGGAGTGGGTTCAGTCTA	643	50.00%
BTV1-WT-S7_RNA-HCR-target_19	tgtgttggattgtgcacatgAAACGTCGGAGTGGGTTCAGTCTA	711	45.00%
BTV1-WT-S7_RNA-HCR-target_20	tatctgaaccatcgcattctAAACGTCGGAGTGGGTTCAGTCTA	733	40.00%
BTV1-WT-S7_RNA-HCR-target_21	tcaaggcagggtattgatttAAACGTCGGAGTGGGTTCAGTCTA	788	40.00%
BTV1-WT-S7_RNA-HCR-target_22	tagttctgttcagtatagccAAACGTCGGAGTGGGTTCAGTCTA	866	40.00%
BTV1-WT-S7_RNA-HCR-target_23	aagatgggtggcagcatattAAACGTCGGAGTGGGTTCAGTCTA	894	45.00%
BTV1-WT-S7_RNA-HCR-target_24	actatctcgatcgtttggtgAAACGTCGGAGTGGGTTCAGTCTA	916	45.00%
BTV1-WT-S7_RNA-HCR-target_25	aacatcagcaagcgtagacaAAACGTCGGAGTGGGTTCAGTCTA	955	45.00%
BTV1-WT-S7_RNA-HCR-target_26	atcgcgaactcaggtcttaaAAACGTCGGAGTGGGTTCAGTCTA	984	45.00%
BTV1-WT-S7_RNA-HCR-target_27	cgtgcaatagcacgtgtgagAAACGTCGGAGTGGGTTCAGTCTA	1032	55.00%
BTV1-WT-S7_RNA-HCR-target_28	cgtgcaaagtggactacacaAAACGTCGGAGTGGGTTCAGTCTA	1061	50.00%
BTV1-WT-S7_RNA-HCR-target_29	cgacacaccgcatatgtaacAAACGTCGGAGTGGGTTCAGTCTA	1088	50.00%
BTV1-WT-S7_RNA-HCR-target_30	gaatgggtcacatatatcccAAACGTCGGAGTGGGTTCAGTCTA	1113	45.00%

Supplementary Table 3. Comparison between computer prediction and SHAPE identified sites

Segment	Computer predicted interacting sites	SHAPE identified sites that are the same or near	Note
**S7**	172-187	172-180	
	233-242	235-242	
	377-388	393-411	Near
	478-489	473-475<br>&<br>493-497	Near
	701-710	705-712	
	1119-1133	1121-1123	
**S8**	85-98	100-107	Near
	459-469	448-451<br>&<br>477-479	Near
	653-663	--	Appear in Stage 2<br>(662-665)
**S9**	28-38	--	Appear in Stage 2<br>(33-35)
	220-229	--	Appear in Stage 2<br>(216-235)
	278-289	274-281	
	428-442	--	Appear in Stage 2<br>(443-445 Near)
	486-495	--	Appear in Stage 2<br>(482-486, 493, 496-499)
	599-613	--	Appear in Stage 2<br>(604-610)
	802-812	--	Appear in Stage 2<br>(800-814)

S10

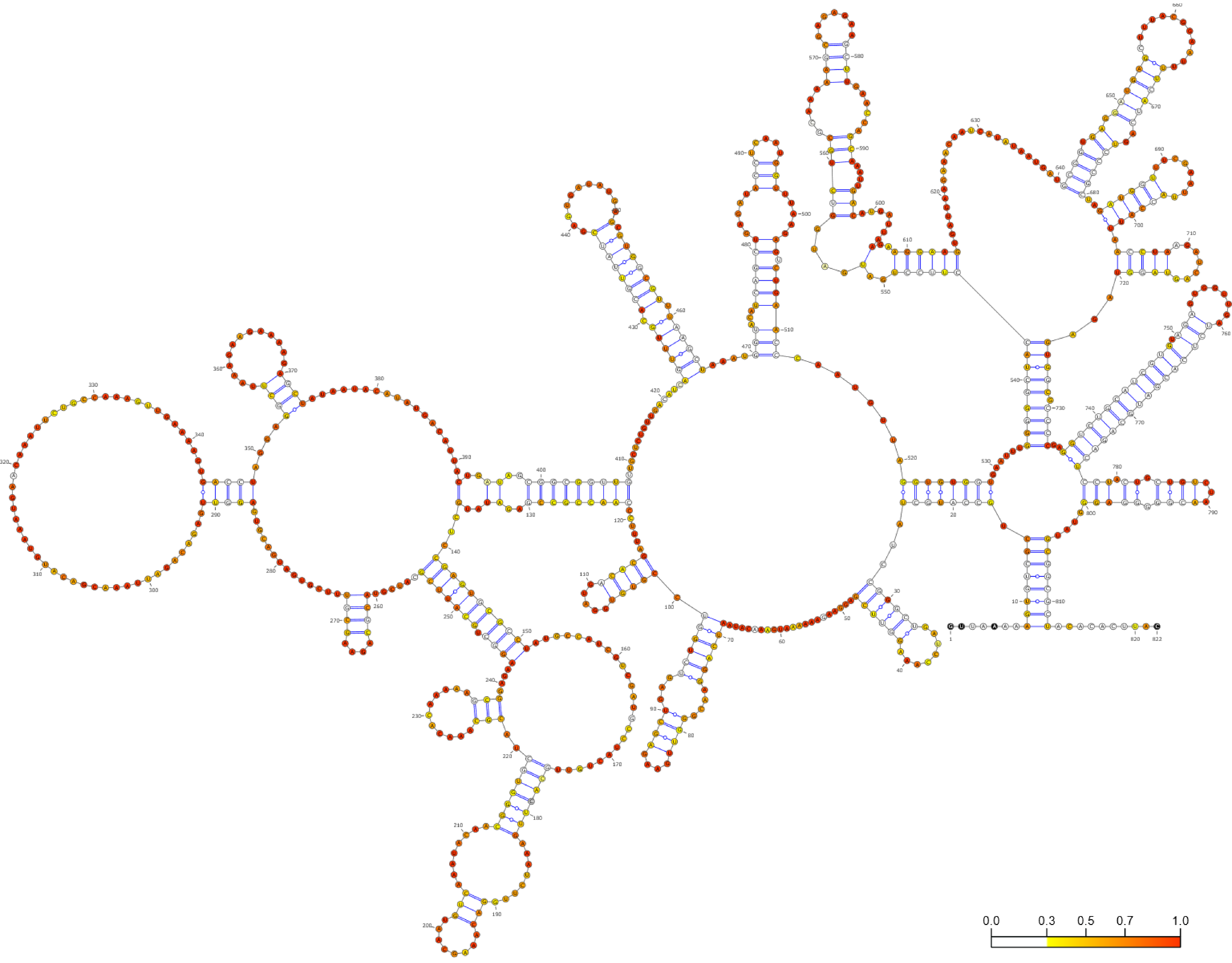

Supplementary Figure 1: Secondary structures of S10, S9, S8 and S7 generated by RNAfold, integrating SHAPE data as a constraint. The strength of SHAPE signals at each nucleotide are shown by different colours as indicated by the colour bar.

S9

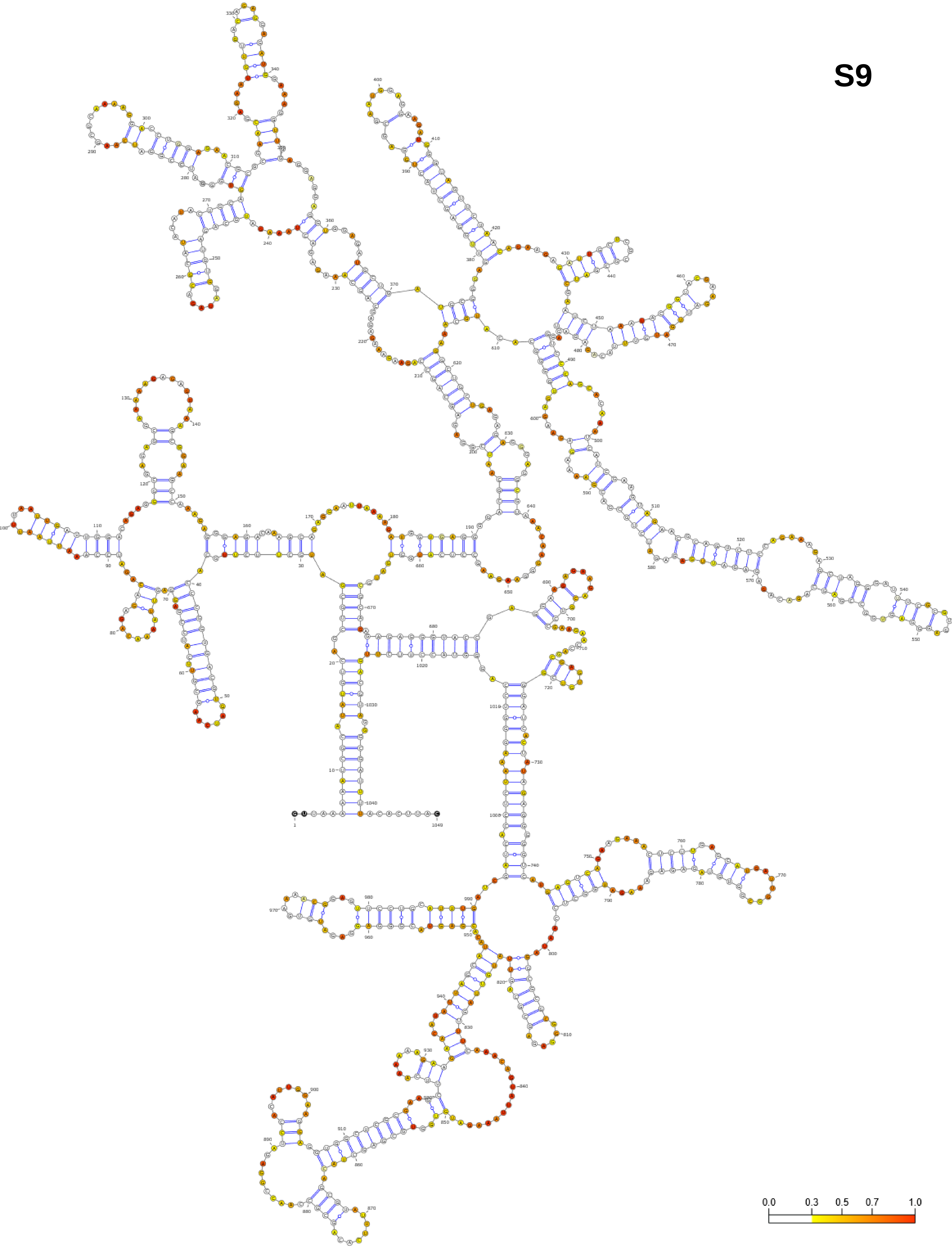

S8

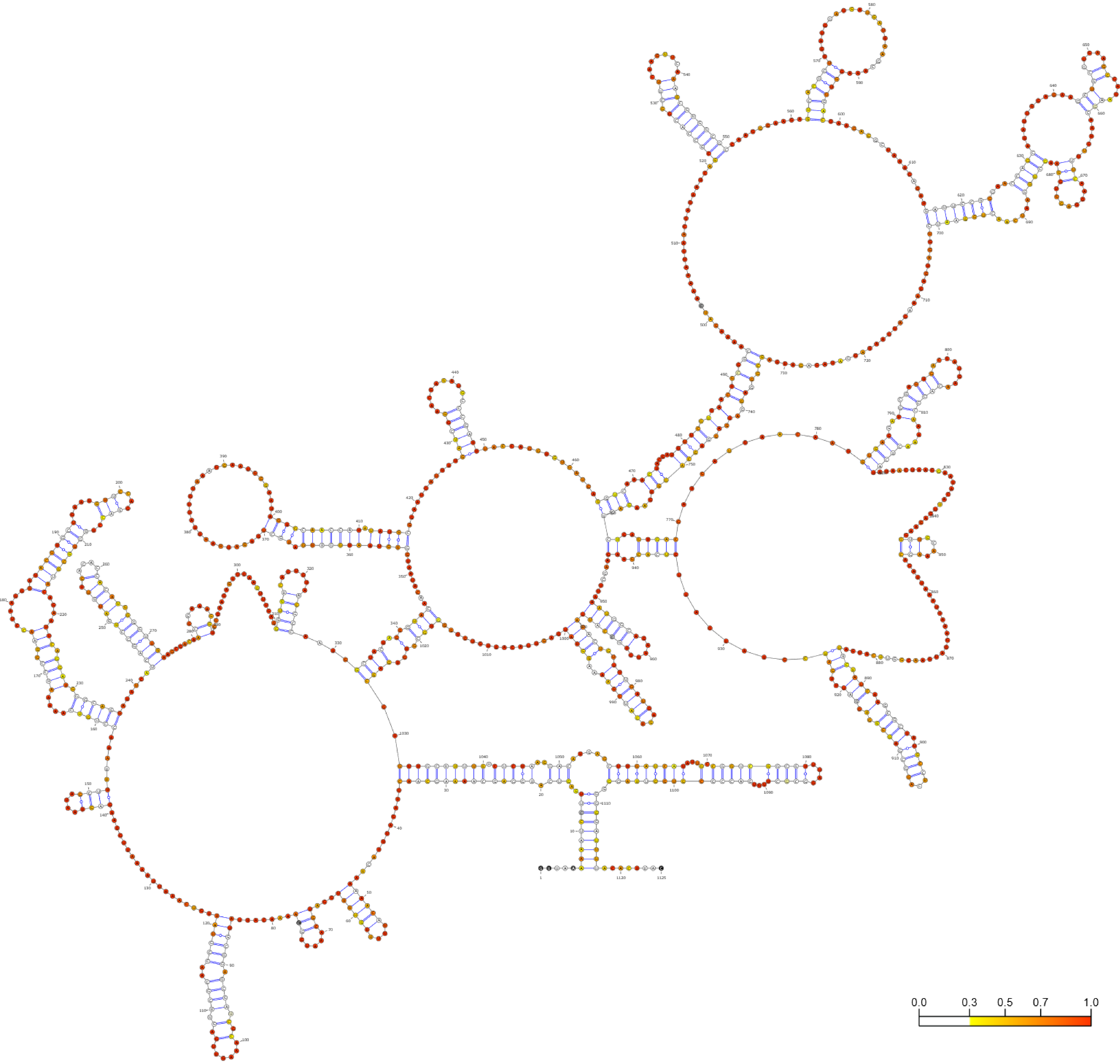

S7

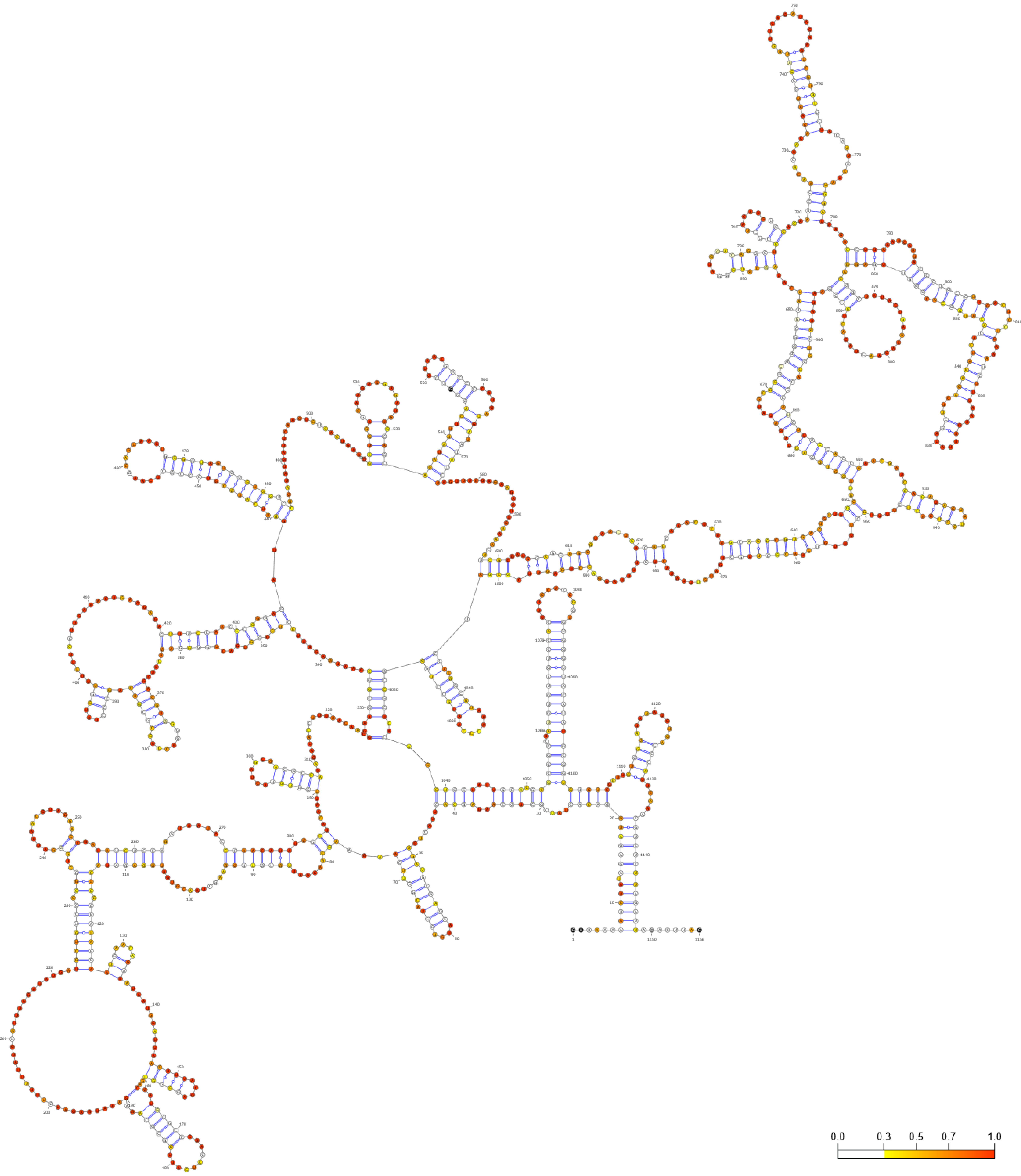

S10

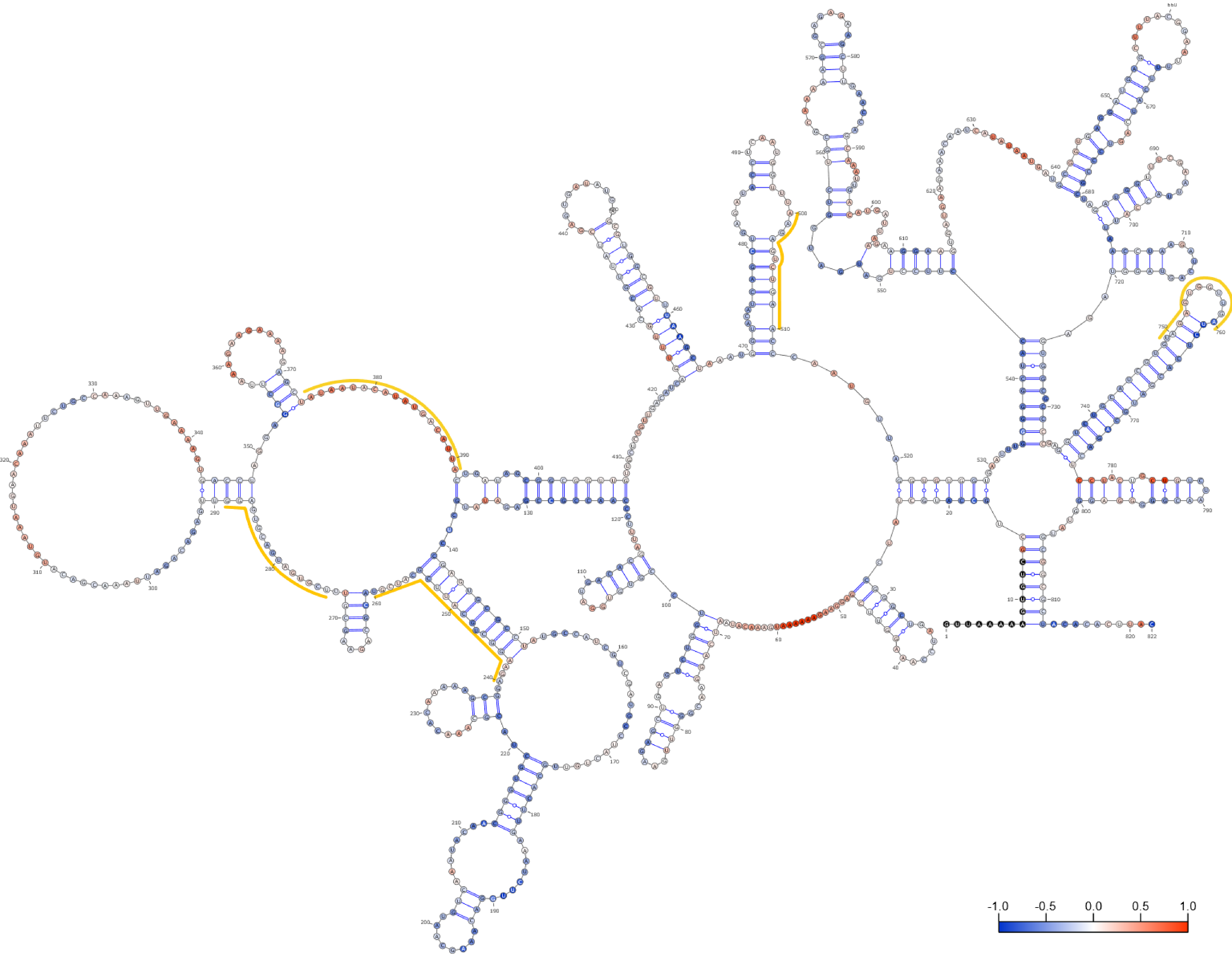

Supplementary Figure 2: Secondary structures of S10, S9, S8 and S7 generated by RNAfold, integrating SHAPE data as a constraint. The ΔSHAPE data of each nucleotide are marked with different colours as indicated by the colour bar (in red and blue). The sites where mutations introduced for the virus recovery assay are also indicated (in yellow).

S9

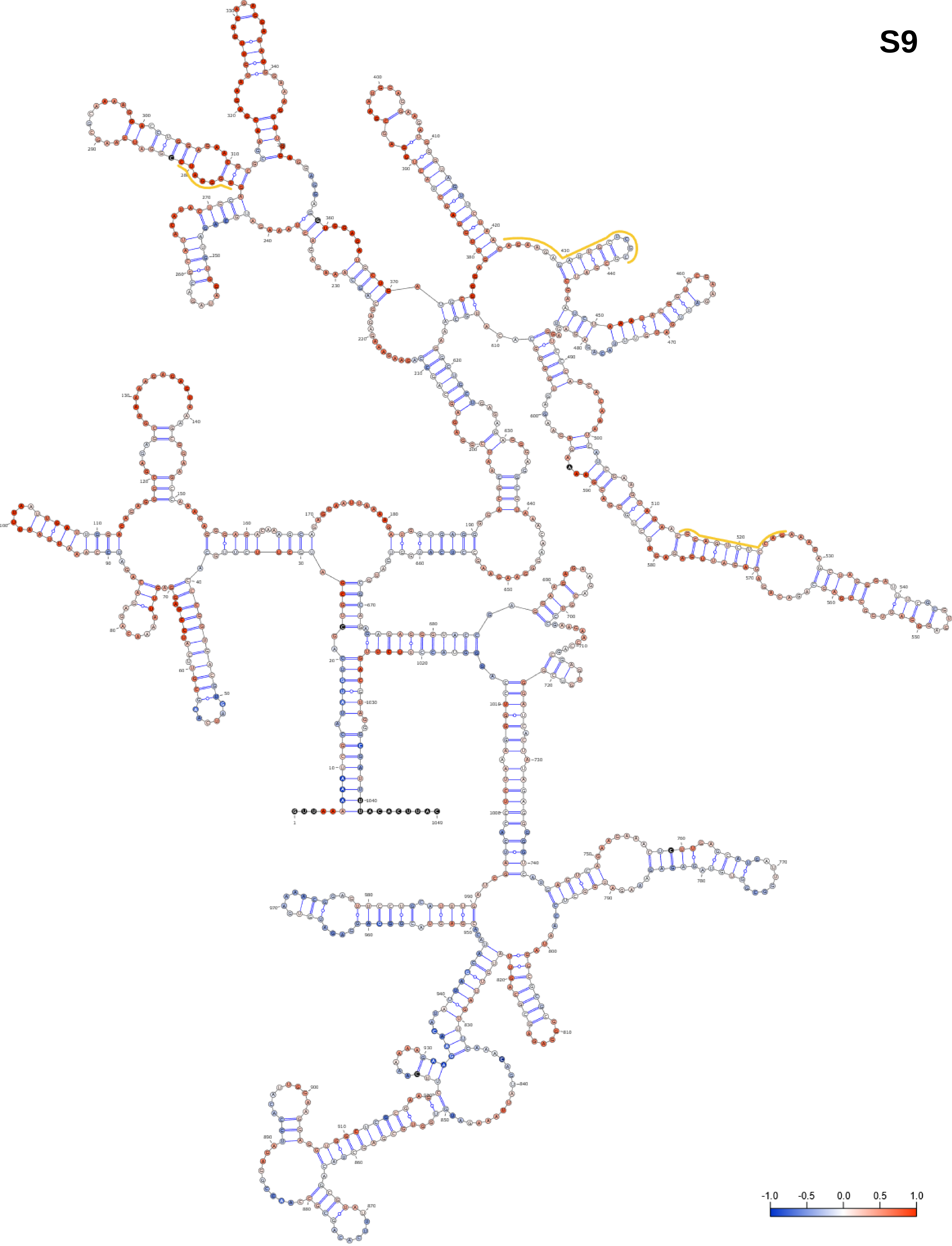

S8

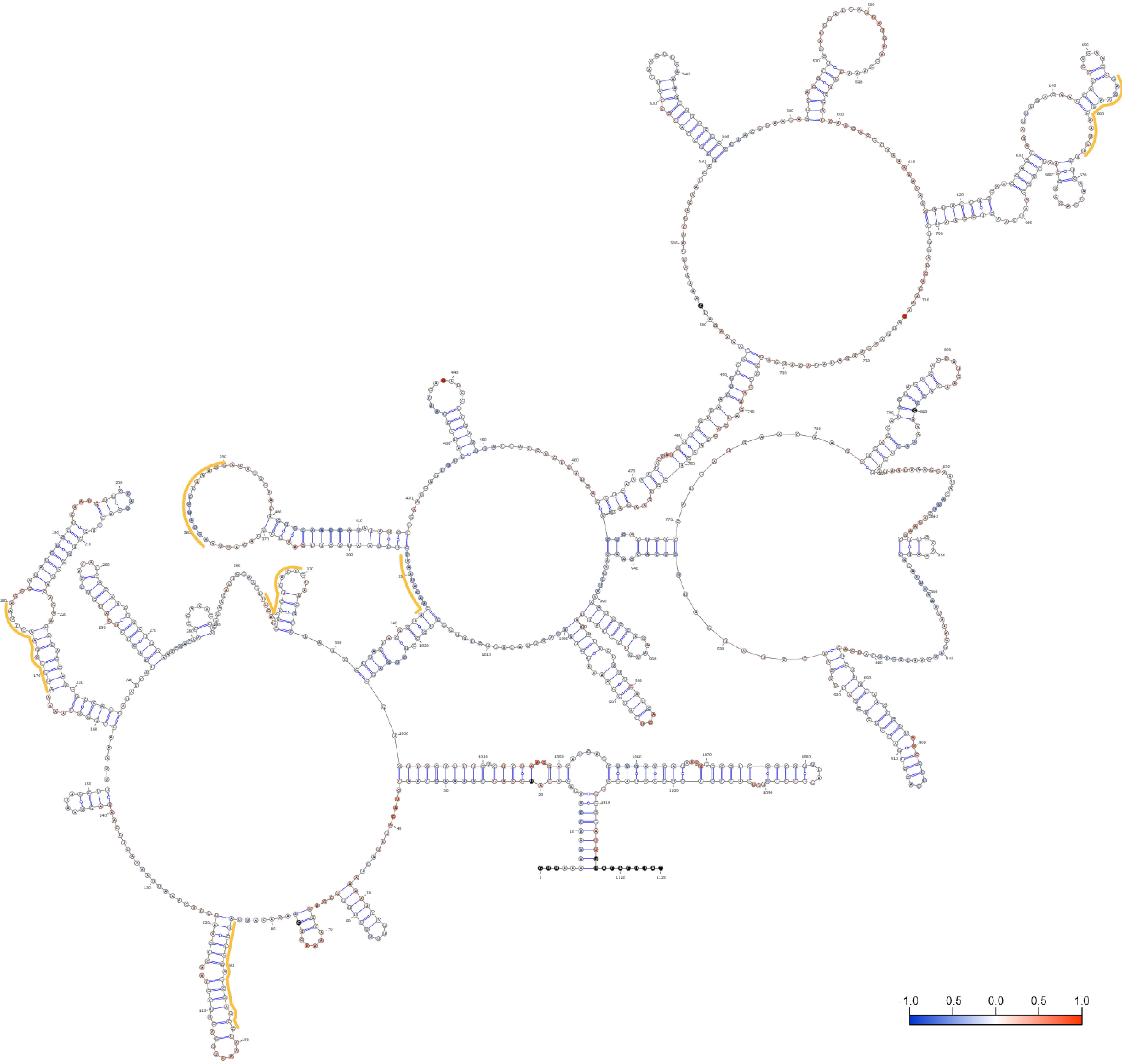

S7

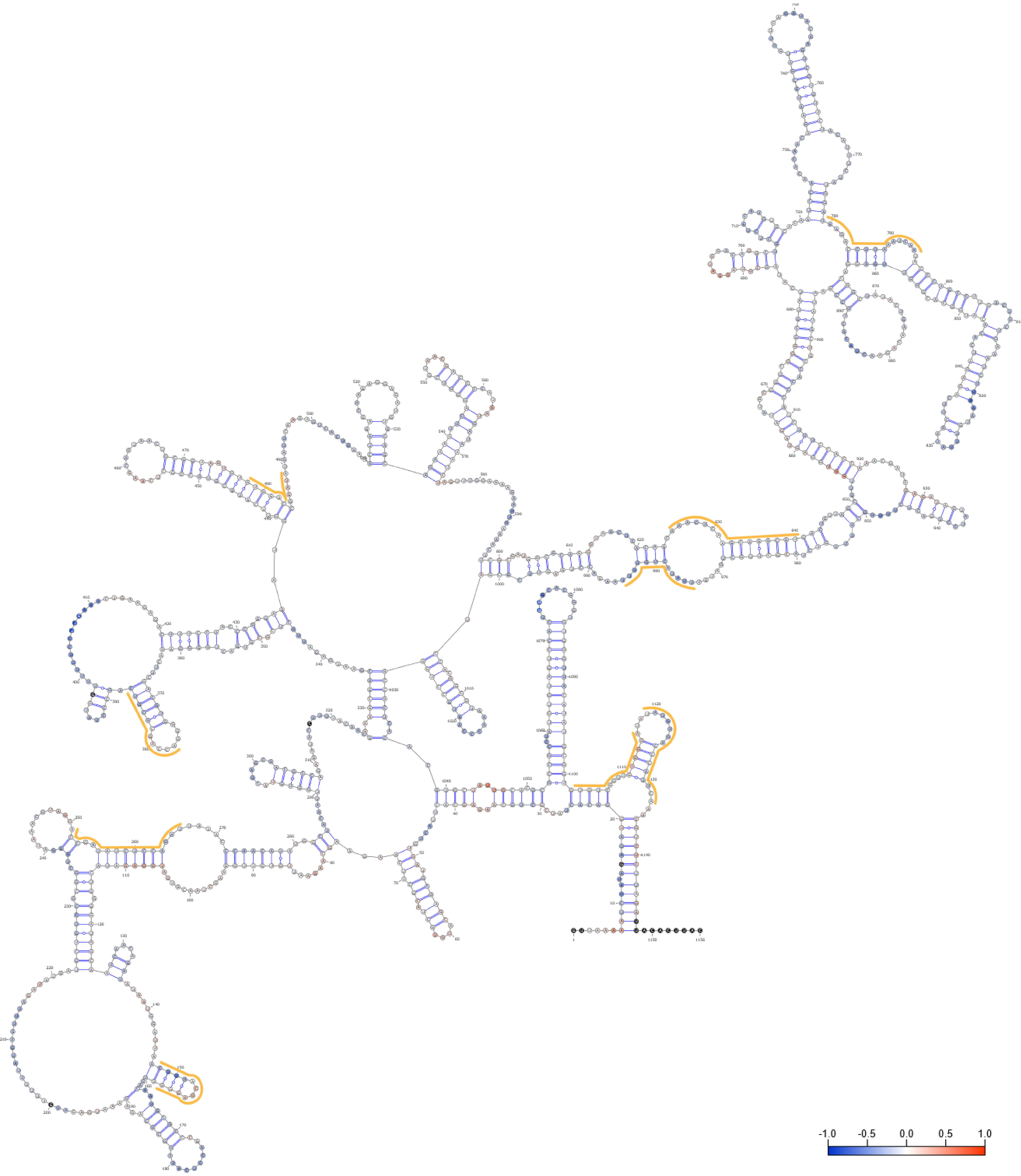

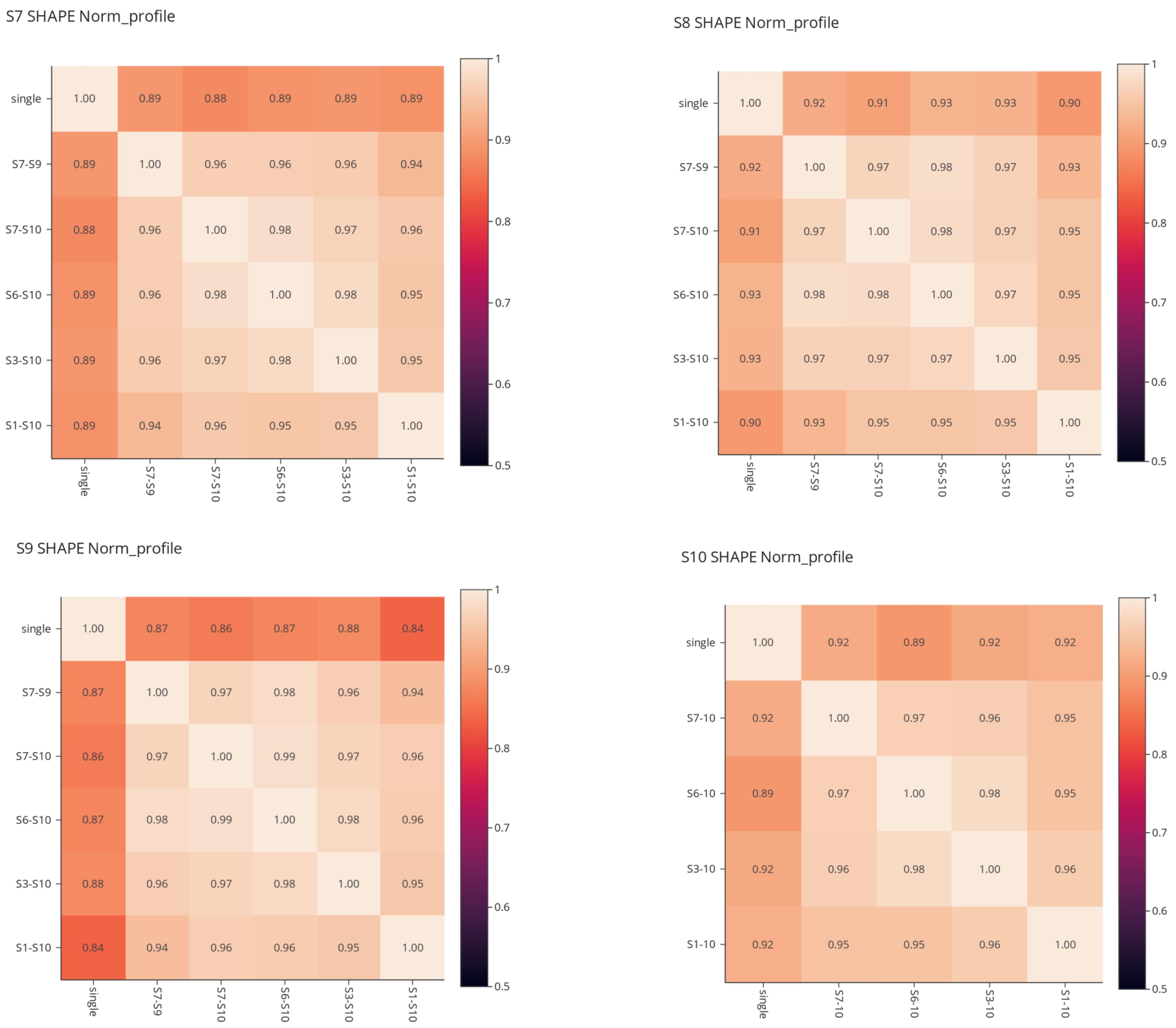

Supplementary Figure 3: Pairwise Pearson correlation coefficients between SHAPE reactivities in different complexes as a measure of similarity. A Pearson correlation coefficient of 1 indicates perfect similarity of SHAPE reactivities.

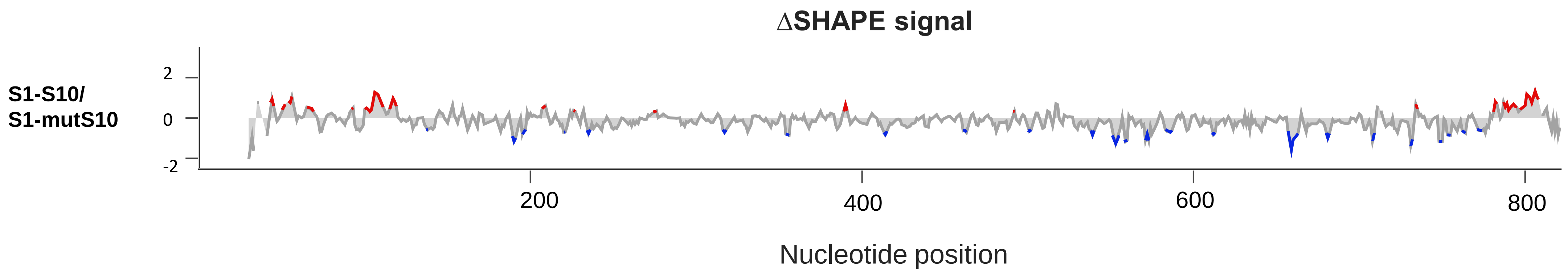

Supplementary Figure 4: The ∆SHAPE profiles of S10 vs mutS10 in the complex with the other nine segments (S1-S9). Line plots showing the ∆SHAPE values indicate the difference in SHAPE values between different complexes S1-S10 and S1- mutS10, where the reactivity values from one complex are subtracted from the other. The red peaks indicate increases of SHAPE signals in the larger complexes and blue valleys indicate decreases.

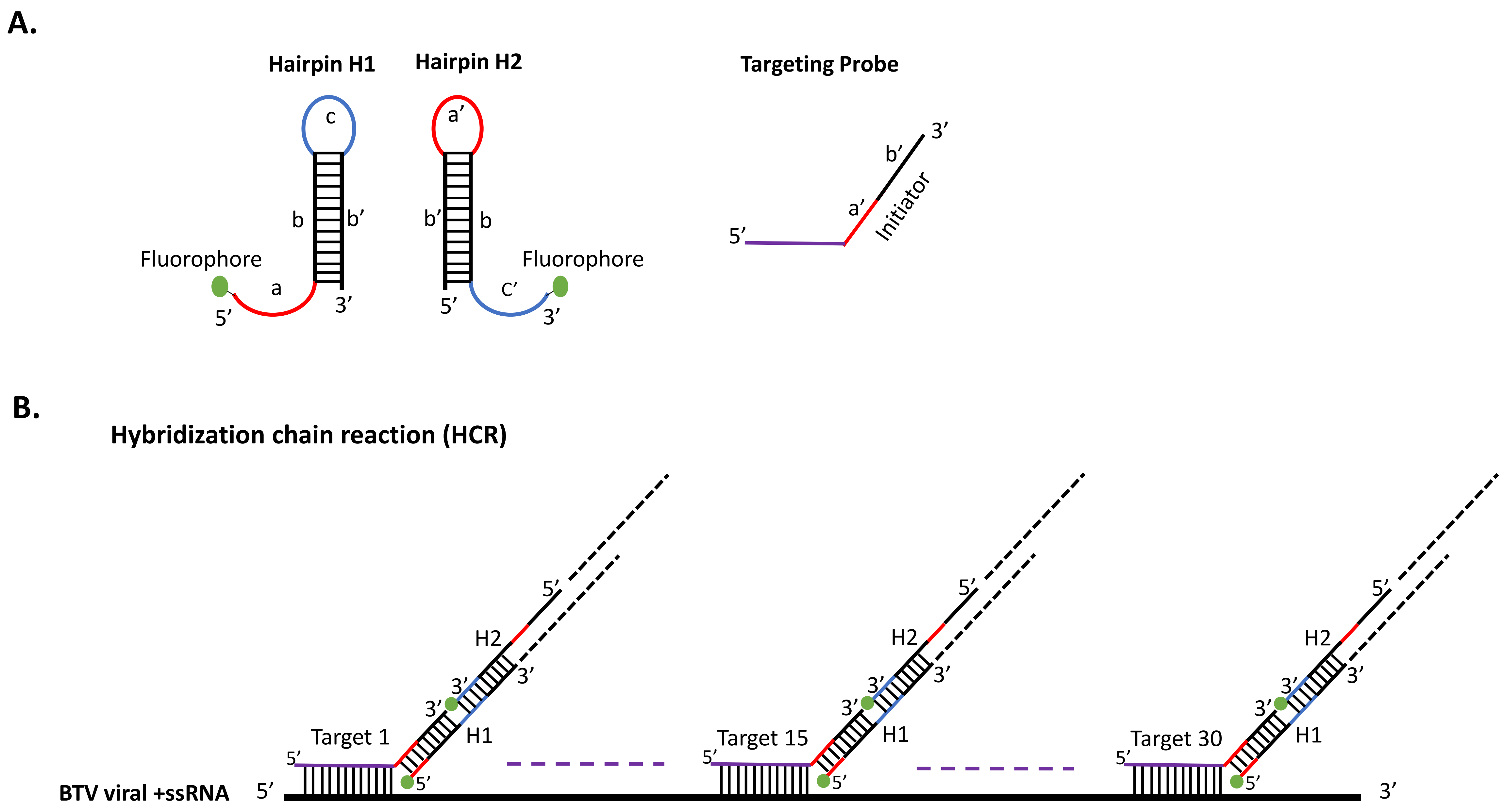

Supplementary Figure 5: A) The structures of H1 and H2 of hairpin DNA pairs, and corresponding targeting probe including initiator sequence. B) The *in situ* hybridisation chain reaction (HCR): 30 targeting probes covered the BTV +ssRNA segment, each of them includes an initiator sequences hybridised to DNA hairpin H1, then, the H1 and H2 continue to hybridise with each other.

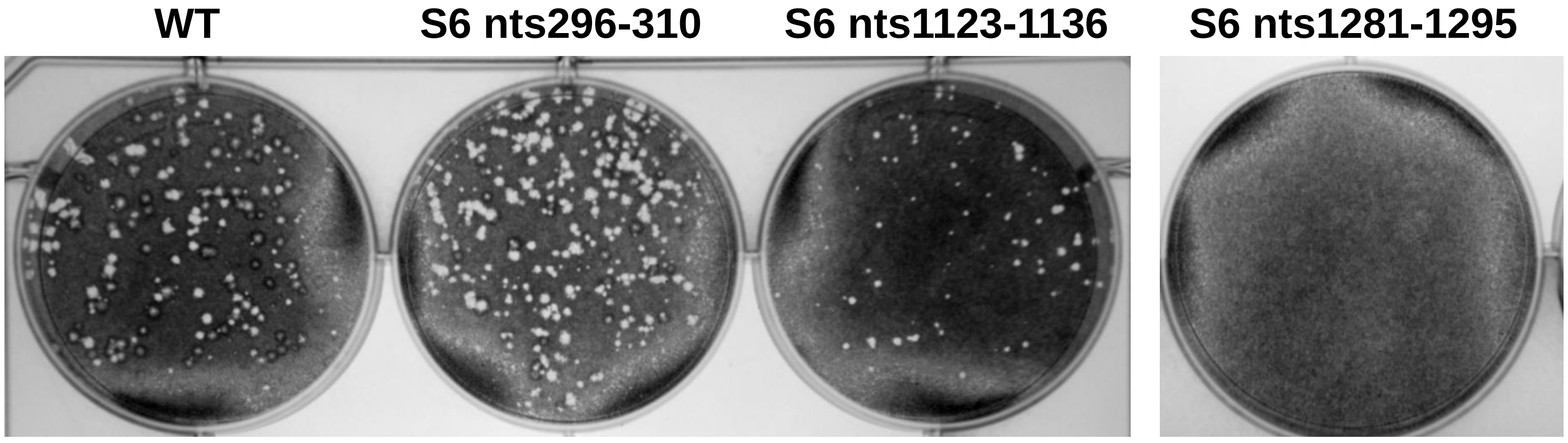

Supplementary Figure 6: The impact of mutating the computer identified RNA interacting sites for virus growth. Mutations were introduced to the indicated RNA interacting sites and RG was performed. Plaques of the virus (if recovered) carrying the mutation are shown
